# Change in the chondroitin/dermatan structure in distal lung tissue from COPD patients

**DOI:** 10.1038/s41598-026-44120-4

**Published:** 2026-03-23

**Authors:** Hani N. Alsafadi, Annika Nybom, Darcy Wagner, Anders Malmström, Sandra Lindstedt, Leif Bjermer, Göran Dellgren, Johan Malmström, Emil Tykesson, Gunilla Westergren-Thorsson, Oskar Hallgren

**Affiliations:** 1 Department of Experimental Medical Science, Lund University, Sölvegatan 19, BMC C12, 22363 Lund, Sweden; 2 Department of Cardiothoracic Surgery, Lund University, Lund, Sweden; 3 Department of Respiratory Medicine and Allergology, Lund University, Lund, Sweden; 4 Department of Cardiothoracic Surgery, The Sahlgrenska Academy, University of Gothenburg, Gothenburg, Sweden; 5 Infection Medicine, Lund University, Lund, Sweden; 6 Trulylabs, Lund, Sweden

**Keywords:** Glycosaminoglycans, Heparan sulfate, Chondroitin/dermatan sulfate, Proteoglycans, Chronic obstructive pulmonary disease, Transforming growth factor-β, Cell biology, Diseases, Medical research, Molecular biology

## Abstract

Chronic obstructive pulmonary disease (COPD) is a progressive condition characterized by airway remodeling, including emphysema and fibrosis. Proteoglycans and their glycosaminoglycan (GAG) chains are key components of the extracellular matrix and may be altered as the disease advances. This study analyzed lung tissue from COPD patients (GOLD stages II–III and IV), non-COPD smokers, and non-smokers to assess proteoglycan and GAG changes. While LC–MS revealed no alterations in chondroitin/dermatan sulfate (CS/DS) or heparan sulfate (HS) proteoglycan core proteins, the total GAG level increased in GOLD II–IV patients. HS displayed increased *N-* and *2-O*-sulfation in GOLD IV, while CS/DS levels and *4-O*-sulfation were enhanced across GOLD II–IV. These findings were supported by transcriptomic data indicating upregulation of CHST11, the main CS/DS *4-O*-sulfotransferase. In line with previous findings, TGF-β signaling was shown to be enriched in COPD patients and to regulate the CHST11 expression. These results were confirmed by TGF-β stimulation of lung fibroblasts showing increased CS/DS levels, *4-O-*sulfation, and CHST11 expression. In conclusion, COPD is associated with disease-stage-specific changes in GAG sulfation, particularly enhanced CS/DS *4-O*-sulfation that is likely to be driven by TGF-β. These alterations may contribute to extracellular matrix remodeling and represent potential targets for therapeutic intervention to mitigate disease progression.

**Supplementary Information:**

The online version contains supplementary material available at 10.1038/s41598-026-44120-4.

## Introduction

Chronic obstructive disease (COPD) is a devastating disorder that is one of the major causes of death globally according to the WHO definition. COPD is characterized by submucosal fibrosis and impaired mucociliary clearance in central and small airways and airway enlargement (emphysema) in the parenchyma, which results in airflow obstruction^1–5^. Tissue remodeling in COPD is characterized by changes in cellular composition but also changes in the extracellular matrix (ECM) composition. Proteoglycans are abundant proteins in the ECM that influence viscoelastic properties, collagen crosslinking, migration, cell differentiation, and act as co-receptors^6^. Their functions are defined both by the core proteins and by the covalently attached linear glycosaminoglycans (GAGs) chains which include chondroitin sulfate/dermatan sulfate (CS/DS), heparan sulfate (HS), keratan sulfate (KS) and hyaluronan (HA). GAGs are negatively charged due to carboxylate and sulfate groups and bind secreted factors including growth factors, cytokines and proteases and matrix metalloproteases (MMPs)^7^. GAG-protein-interactions are not only dependent on molecular charge but also on the positions of sulfate groups and the carbohydrate structure creating distinct binding motifs^8,9^. While several studies have paid attention to proteoglycans in COPD, less is known about GAGs and their fine structure such as sulfation/epimerization/acetylation. The synthesis and modification of GAGs are carried out by a number of endoplasmic reticulum (ER) and Golgi-resident enzymes^10^. The first step of the biosynthesis is formation of a tetrasccharide linkage region on serine residues on the core protein by xylosyl-transferase XYLT1-2, galactosyltransferases (B3GAL6-7) and uronosyltranferase (B3GAT3), a process that is identical for both HS and CS/DS synthesis. The HS biosynthesis continues with addition of GlcNAc by EXTL1-2. This is followed by extension of the chain by addition of alternating GlcA and GlcNAc residues which is carried out by the polymerases EXT1-2. Simultaneously, the GlcNac residues are partially N-deacetylated and N-sulfated by NDST1-4. Some GlcA residues are next epimerized to IdoA by the HSC5 epimerase GLCE, followed by *2-O* sulfation by HS2ST2 of some uronic acid residues. Final modifications include *3-O* sulfation by HS3ST1-6 and *6-O* sulfation of HS6ST1-3. Functionally, *3-O* sulfation is of importance as it is present in most known specific motifs for protein ligands, including the antithrombin motif in heparin. The first step in the CS/DS biosynthesis is the addition of a GalNac residue to the linkage region by CSGALNACT1-2 followed by polymerization by a family of polymerases, including CHPF and CHSY1, that adds alternating GlcA and GalNac residues. The non-sulfated GlcA-GalNac unit serves as substrate for *4-O* sulfotransferases, CHST11-13, and *6-O* sulfotransferases CHST3 and CHST7 that adds sulfate groups to GalNac residues. GlcA-GalNac(*4-O* sulfate) can be further sulfated by CHST15 resulting in GlcA-GalNac (*4, 6-O*-disulfate). GlcA residues can be epimerized by DSE and DSEL that converts GlcA to its stereoisomer IdoA. The presence of IdoA residues in the polymer defines DS and the IdoA content varies from a few percent up to almost 100 percent. The DSE and DSEL activity is tightly linked to the *4-O* sulfotransferase CHST14 and the *2-O* sulfotransferase UST that mainly add sulfate groups to IdoA-GalNac units.

The expression of GAGs has not been fully characterized in the context of COPD but alterations have been reported in a variety of diseases such as atherosclerosis, cancer, diabetes, neurodegenerative diseases, virus infection, lung fibrosis and asthma^11^. The aim of the present study was to characterize proteoglycans and GAGs and their regulation in lung tissue from COPD patients with varying severity.

## Results

### Proteoglycan levels and expression in COPD lung tissues

**Fig. 1:**
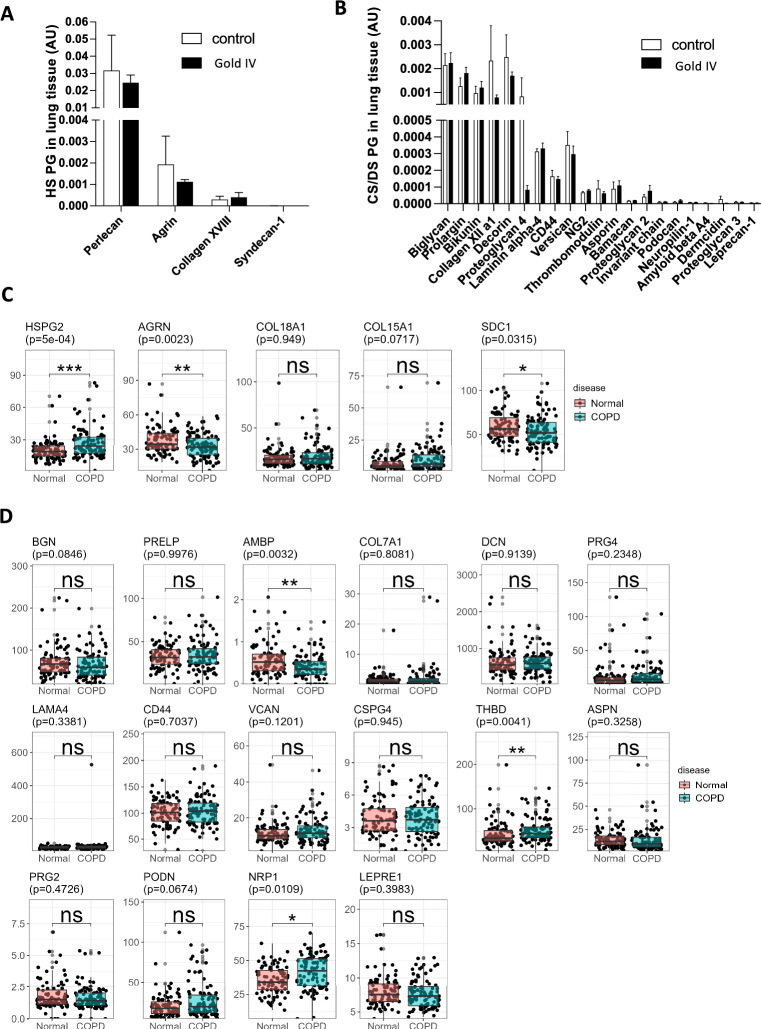
Proteoglycan levels and expression in COPD lung tissues. Proteomic quantification of proteoglycans in distal lung tissue from controls (n = 5) and COPD GOLD stage IV patients (n = 5) (**A**) HS proteoglycans and (**B**) CS/DS proteoglycans. Data is presented as mean ± S.D. Transcriptomic analysis in distal lung tissue from controls (n = 91) and COPD patients (n = 95) (**C**) heparan sulfate proteoglycans and (**D**) CS/DS proteoglycans. * adj.*p*-value < 0.05, ** adj.*p*-value < 0.01 and *** adj.*p*-value < 0.001 using empirical Bayes analysis (limma) and adjusted with FDR via Benjamini-Hochberg. Reported *p*-value in plot title is the adjusted-*p*-value.

An examination of the matrisome proteome in distal lung tissue from controls (n = 5) and GOLD stage IV COPD patients (n = 5) has previously been reported^1^. The dominating HS proteoglycan was perlecan (Fig. 1A) while smaller amounts of the basement membrane proteoglycans agrin, collagen XVIII, collagen XV and syndecan 1 was found in controls and COPD patients. The CS/DS proteoglycans, biglycan, decorin, collagen XII, bikunin, proteoglycan 4 (lubricin) were the most abundant proteoglycans in lung tissue from controls and COPD patients (Fig. 1B), while laminin alpha 4, versican and CD44 were present in smaller amounts. There were no statistically significant changes observed in this limited data set due to the low number of patients utilized. Therefore, we sought to explore these changes in a clinical dataset with larger patient numbers but with less severe disease (GSE57148: 98 COPD GOLD stage I-II patients and 91 subjects with normal spirometry). We extracted transcriptional levels of proteoglycans and found that changes in expression levels of HS proteoglycans followed similar patterns as their protein expression except for perlecan, which was increased (Fig. 1C). In addition, CS/DS-proteoglycan gene expression exhibited similar trends as to their protein expression except for bikunin (AMBP) which decreased in COPD patients and versican (VCAN) and thrombomodulin (THBD) that increased (Fig. 1D).

### Total glycosaminoglycans are increased in COPD with structural changes in CS/DS and HS

**Fig. 2:**
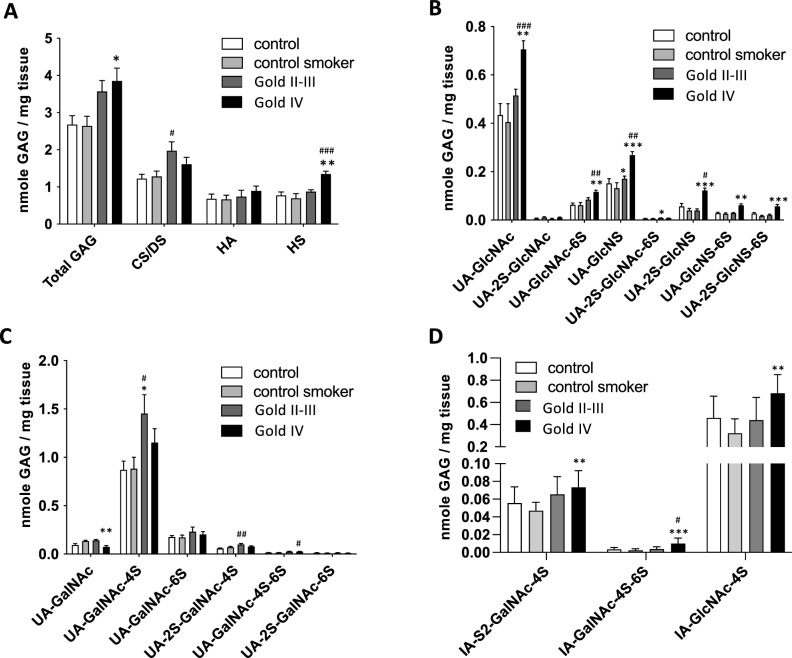
GAG analysis in COPD lung tissues. Quantification of GAGs in distal lung tissue from controls (n = 11), control smokers (n = 11) and COPD patients in GOLD stage II-III (n = 11) and GOLD stage IV (n = 11). (**A**) Total GAG, CS/DS, HA and HS, was quantified as the sum of relevant AMAC-derivatized disaccharides. (**B**) Quantification of HS disaccharides, (**C**) Quantification of CS/DS disaccharides, (**D**) Quantification of DS disaccharides following chondroitinase B digestion. Due to limited tissue supply, there were only 9 subjects in the control group and in the GOLD stage IV group for this analysis. # comparison to controls, * comparison to control smokers. Data is presented as mean ± S.D., *p < 0.05, **p < 0.01 and ***P < 0.001 using Kruskal–Wallis tests combined with Dunn’s multiple comparison test.

The total GAG content, i.e. the sum of CS/DS and HS and HA, was significantly increased in lung tissue from GOLD stage IV patients compared to control smokers (3.9 ± 1.1 vs 2.6 ± 0.9 nmole GAG/mg tissue) (Fig. 2A). No differences between controls and control smokers were observed. The dominating GAG was CS/DS that constituted approximately 50% of the total GAG fraction. It was signifcantly increased in GOLD stage II-III patients compared to controls (2.0 ± 0.8 vs 1.2 ± 0.4 nmole GAG/mg tissue) (Fig. 2A). HS constituted approximately 30% of the total GAG fraction and was significantly increased in GOLD stage IV patients compared to controls and control smokers (1.3 ± 0.3 vs 0.9 ± 0.2 and 0.7 ± 0.4 nmole GAG/mg tissue, respectively). HA made up approximately 20% of the total GAG fraction and was not changed in COPD patients compared to the control groups. The most abundant HS disaccharide was UA (Uronosyl)-GlcNAc followed by UA-GlcNS and UA-GlcNAc-6S (Fig. 2B, supplemental Fig. S1). Most *N-*sulfated and *2-O*-sulfated disaccharides were increased in GOLD stage IV patients compared to controls (Fig. 2B). We were not able to analyze *3-O* sulfated HS disaccharides due to the lack of commercially available standards. This modification is rare but contributes to the structural and functional specificity of HS by its ability to bind cytokines and coagulation factors. The most abundant CS/DS disaccharide was UA-GalNAc-4S that was significantly increased in GOLD stage II-III compared to controls and control-smokers (1.5 ± 0.6 vs 0.9 ± 0.3 and 0.9 ± 0.4 nmole GAG/mg tissue, respectively) (Fig. 2C, supplement Fig. S2). A common modification of chondroitin sulfate is the epimerization of glucuronic acid (GlcA) to iduronic acid (IdoA) in dermatan sulfate and has been demonstrated to have functional implications^12^. The IdoA-GalNAc-4S content was approximately 50% of the total UA-GalNAc-4S and was significantly increased in GOLD stage IV patients (Fig. 2D). In controls, control-smokers and GOLD stage II-III patients the IdoA-GalNAc content was approximately 40% of total *4-O*-sulfated disaccharides. IdoA-2S-GalNAc-4S was significantly increased in GOLD stage II-III patients compared to control-smokers, while IdoA-GalNAc-4S,6S was significantly increased in GOLD stage IV patients compared to controls and control-smokers.

### CHST11 expression is increased in COPD lung tissues

**Fig. 3:**
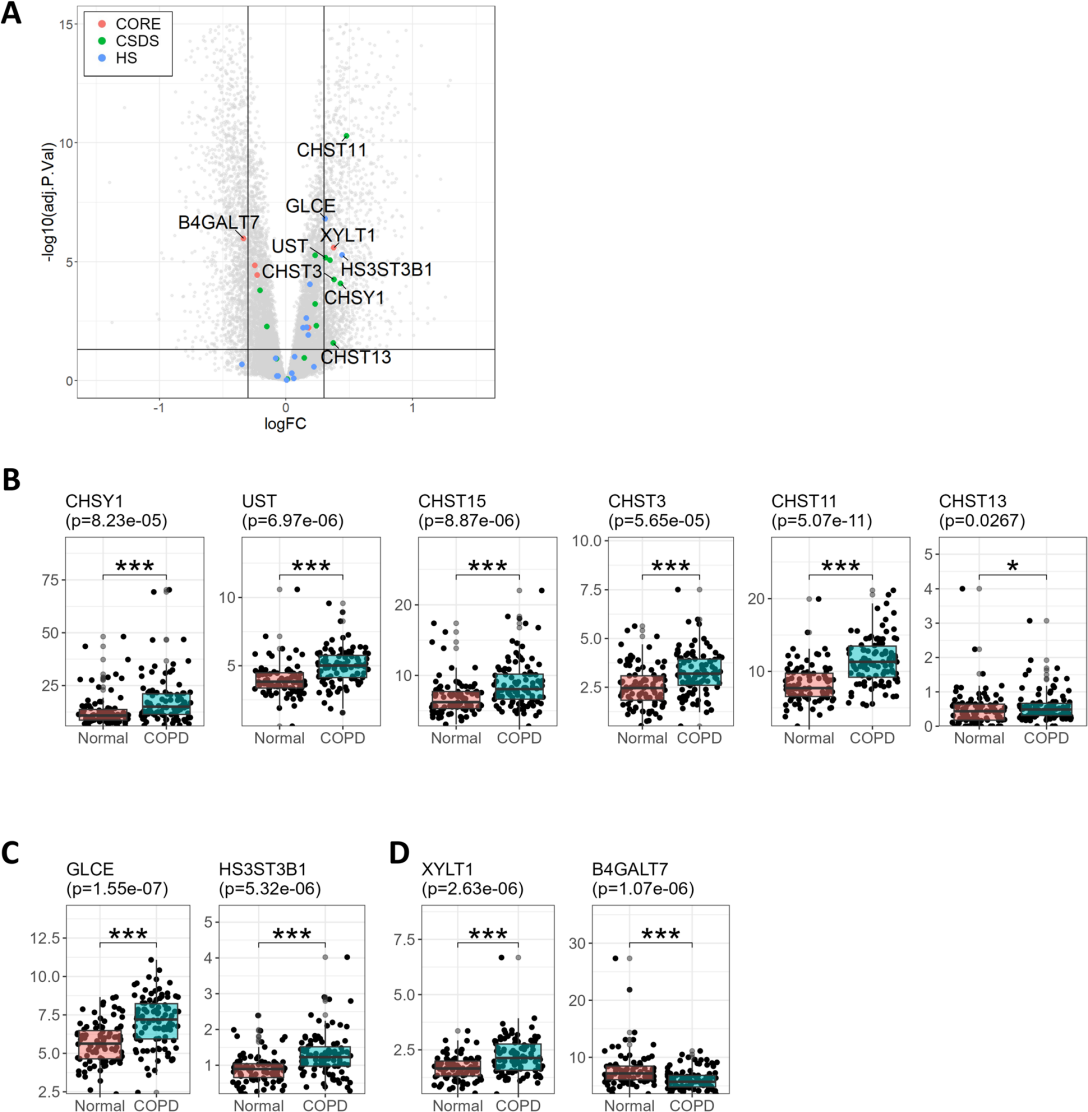
Expression of biosynthetic GAG enzymes in COPD lung tissues. Gene expression profiling of COPD tissues from cohort 2 using RNAseq. (**A**) Volcano plot of the differential expression of COPD (GOLD stage I-II, n = 96) versus. control tissues (n = 91) highlighting the genes corresponding to the biosynthetic enzymes of the glycosaminoglycans. Cut-offs: −0.3 < log Fold Change < 0.3; adj.*p*-value < 0.05. (**B**) Gene expression of CS/DS-related biosynthetic genes in FPKM, (**C**) Gene expression of HS-related biosynthetic genes in FPKM, (**D**) Gene expression of core related genes. * adj.*p*-value < 0.05, ** adj.*p*-value < 0.01 and *** adj.*p*-value < 0.001 using empirical Bayes analysis (limma) and adjusted with FDR via Benjamini-Hochberg. Reported p-value in plot title is the adjusted-*p*-value.; Genes of other biosynthetic enzymes are in supplemental figure S3.

To validate the results and further examine the increase and modification in HS and CS/DS synthesis, the gene expression of the GAG biosynthetic enzymes was explored in a previously published RNAseq dataset, GSE57148, of normal smokers and COPD GOLD stage I-II patient tissues from distal lung (Fig. 3). COPD patients in this cohort had slightly higher FEV1 predicted than the GOLD II-III patients used for the GAG analysis, but pack years and age were well matched. Differential expression analysis showed increased expression of several of the biosynthetic and modifying enzymes especially for CS/DS and HS, while several of the core related enzymes were downregulated (Fig. 3A and supplemental Fig. S3C). In agreement with the structural data of CS/DS, there was a significant upregulation of several enzymes responsible for the synthesis and modification of CS/DS polysaccharides such as the *4-O*-sulfotransferases CHST11, CHST13, the *6-O*-sulfotransferases CHST3, CST15, the uronosyl*-2-O*-sulfotransferase (UST) and the polymerase CHSY1 in COPD patients compared to control subjects (Fig. 3B). This was also accompanied by a small upregulation in expression of the epimerases DSE and DSEL, which are important for the epimerization of glucuronic acid to iduronic acid and thus in the conversion of CS to DS (Supplemental Fig. S3A). Furthermore, a smaller number of HS biosynthetic and modifying enzymes were significantly upregulated such as the C5 epimerase GLCE and the* 3-O-*sulfotransferase HS3ST3B1 (Fig. 3C and supplemental Fig. S3B). In addition, one of the GAG core structure enzymes, XYLT1, was upregulated, while several other core related enzymes were downregulated (Fig. 3D and Supplemental Fig. S3C). Among all the GAG biosynthetic enzymes, CHST11 was the most significantly upregulated between control subjects and COPD patients (Fig. 3A and B).

### CHST11 expression in distal lung tissue

**Fig. 4:**
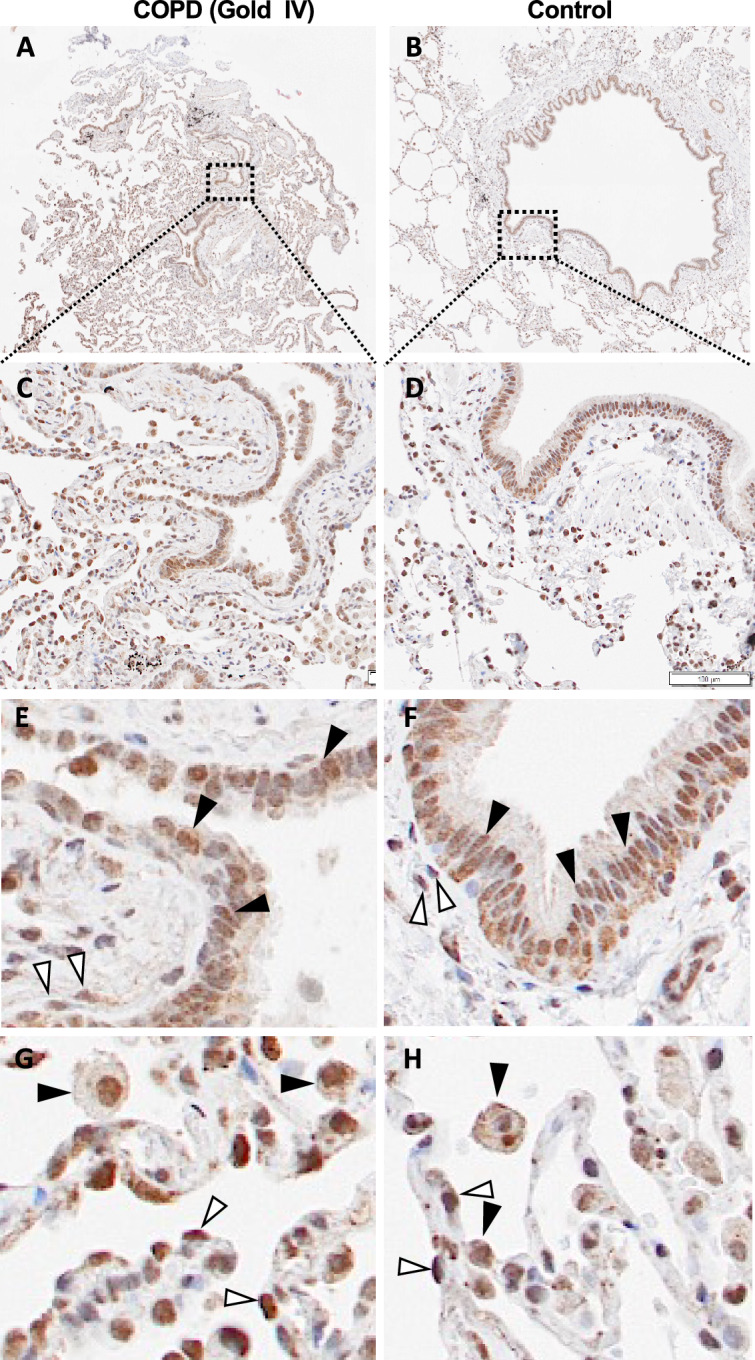
Immunohistochemistry for CHST11 in in lung tissues. Immunohistochemistry for CHST11 in lung tissue from one COPD GOLD stage IV patient (**A**), (**C**), (**E**) and (**G**) and from one control subject (**B**), (**D**), (**F**) and (**H**). (**E**) and (**F**), black arrowheads show immunopositive bronchial epithelial cells and white arrowheads immunopositive peribronchial fibroblasts. (**G**) and (**H**), black arrowheads show immunopositive alveolar macrophages cells and white arrowheads immunopositive alveolar type 2 cells.

We used immunohistochemistry to examine which cell types that expressed CHST11 in distal lung tissue from one control and one COPD GOLD stage IV patient (Fig. 4). As expected, CHST11 was found in proximity to the cell nuclei in most cell types as it is a Golgi-resident protein. Immunopositivity for CHST11 was observed in a majority of bronchial epithelial cells, peribronchial fibroblasts, alveolar type II cells and alveolar macrophages while less staining was observed in vascular structures and in smooth muscle cells. The most intense staining was found in alveolar type II cells. There was no difference in the cellular staining profile between control and COPD tissues.

### TGF-β stimulation recapitulates changes in GAG production and modification observed in distal lung tissue from COPD patients

**Fig. 5:**
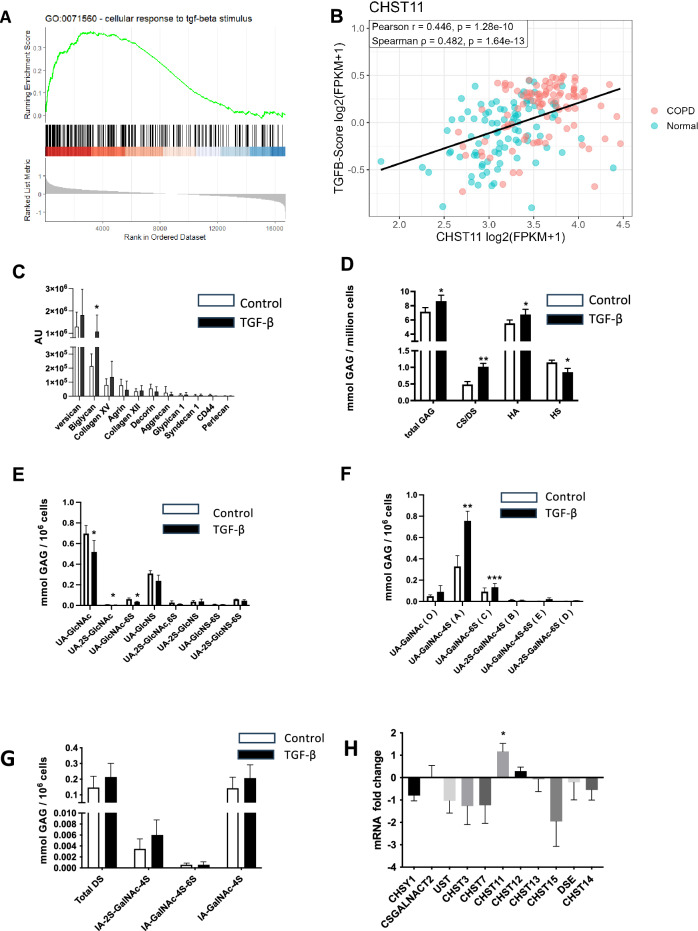
Effect of TGF-β activity. (**A**) Gene set enrichment analysis (GSEA) of ranked list generated from differential expression of RNAseq data from cohort 2 was performed on COPD patients (GOLD stage I-II, n = 96) versus controls (n = 91) lung tissue enriched against cellular response to TGF-β stimulus. (**B**) Pearson and Spearman correlation between CHST11 and TGF-β score generated using enrichment scoring for each sample. Correlation of all GAG enzymes are found in Supplemental Fig. S5. (**C**) Quantification of proteoglycans in HFL1 cells following TGF-β stimulation measured using SRM technology with pre-chosen peptides described in supplemental Table 1, (n = 4). (**D**) Quantification of GAGs in HFL1 cell medium following TGF-β stimulation (n = 4). (**E**) Quantification of HS disaccharides in HFL1 cell medium following TGF-β stimulation (n = 4). (**F**) Quantification of CS/DS disaccharides in HFL1 cell medium following TGF-β stimulation, (n = 4). (**G**) Quantification of DS disaccharides in HFL1 cells following TGF-β stimulation using AMAC-labelling after chondroitinase B digestion (n = 4). (**H**) mRNA fold change of selected CS/DS biosynthetic enzymes in TGF-β stimulated HFL1 cells compared to vehicle control measured by qPCR, (n = 4). Data is presented as mean ± S.D., *p < 0.05, **p < 0.01 and ***P < 0.001 using Student’s T-test.

The regulation of the production and modification of CS/DS GAGs is complex as it is not template driven and relies on the coordinated effort of multiple enzymes and molecular interactions for controlled and targeted posttranslational modification. However, more is known about the stimuli which regulates these enzymes and one stimulus that has been shown to influence CHST11 expression is TGF-β^13–15^. Therefore, we performed gene set enrichment analysis (GSEA) on the differentially expressed genes from the COPD cohort, GSE57148, to examine whether known TGF-β targets were enriched. We found that the upregulated genes in the COPD transcriptome were enriched with known TGF-β targets (Fig. 5A), indicating the ability of TGF-β to drive some of the observed effects. Furthermore, to evaluate which biosynthetic GAG enzymes that were specifically correlated with TGF-β signaling, we generated a TGF-β score for each sample in the RNAseq using enrichment scoring for each sample using Gene Set Variation Analysis (GSVA) and evaluated how the gene expression of each GAG enzyme correlated with the TGF-β score using Pearson and Spearman correlations (Fig. 5B and Supplemental Fig. S5). Interestingly, we found CHST11 to have a moderate but significant positive correlation with the TGF-β score (Fig. 5B). In addition, several CS/DS polymerization and modifying enzymes responsible for initiation, polymerization, and modification of the CS/DS chain such as CHST3, CHST15, CSGALNACT1, CSGALNACT2, CHSY1, UST, DSE, and DSEL showed a significantly positive correlation with varying degree of correlation (Supplemental Fig. S5A). These analyses indicate that the CD/DS modifications observed in COPD are correlated with the TGF-β expression profile in this patient cohort.

As fibroblast was one of the cell types that were immunopositive for CHST11, we used human fetal lung fibroblasts (HFL1) and stimulated them with TGF-β to mechanistically link TGF-β to the structural changes in GAGs observed in lung tissue. Using SRM technology and LC/MS it was observed that versican, biglycan, collagen XV, agrin, collagen XII and decorin were the predominant proteoglycans produced by HFL1 cells (Fig. 5C). TGF-β induced a fivefold increase of biglycan but no other proteoglycans were significantly changed. GAG disaccharide analysis revealed that TGF-β increased CS/DS two-fold (Fig. 5D), which agrees with the GAG composition observed in lung tissue from GOLD stage II-III and IV patients (Fig. 2A). TGF-β treatment decreased HS while HA was increased which contrasted to what was observed in COPD lung tissue. (Fig. 5D and Fig. 2A). TGF-β decreased the major HS disaccharide UA-GlcNAc and UA-GlcNAc-6S (Fig. 5E). The TGF-β induced increase of CS/DS was mainly due to a 2.3-fold increase in UA-GalNAc-4S and to a smaller extent to an increase in UA-GalNAc-6S while other disaccharides were unaffected by the treatment (Fig. 5F). A tendency to increased IdoA content in CS/DS was observed after TGF-β stimulation which is in accordance with that observed in lung tissue from GOLD stage IV patients (Fig. 5G and Fig. 2D). Finally, the effect of TGF-β on the expression of selected sulfotransferases, polymerases and epimerases in CS/DS synthesis was examined using qPCR. CHST11 was significantly increased by TGF-β which validates the structural and expression data from COPD patients (Fig. 5H).

## Discussion

We observed an increase in CS/DS in lung tissue from COPD GOLD stage II-III patients. The most abundant disaccharide in CS/DS in distal lung tissue was UA-GalNAc-*4-O*-S that constituted almost 80% of the CS/DS polysaccharide. This specific CS modification is catalyzed by the *4-O*-sulfotransferases CHST11, CHST12 and CHST13 but CHST11 is by far the most abundant in lung tissue. In the RNA sequencing dataset, there was an increase in both CHST11 and CHST13 in COPD patients compared to controls. CHST11 has been shown to be increased and contribute to progression of lung cancers characterized by tissue remodeling, including lung adenocarcinoma and non-small-cell lung cancers as well as in lung fibrosis^16^. A common risk factor for lung cancer, lung fibrosis and COPD is cigarette smoking, and one might consider a possible correlation between cigarette smoking and the increase in CHST11 and CHST11-driven modifications in CS/DS oligomers^17^. We did not observe a difference in the known CHST11-driven modifications between non-smokers and smokers, which suggests that cigarette smoking history per se is not sufficient to induce such modifications and that there may be additional disease specific factors that work in concert in the disease setting.

TGF-β is one factor that has been shown to influence CS/DS production and modification and especially the *4-O-*sulfation by inducing the expression of CHST11^13^. There is evidence that this connection is relevant in lung cancer and pulmonary fibrosis^16^. Treatment of lung fibroblasts with TGF-β in the current study increased CS/DS and in particular UA-GalNAc-4S and CHST11 expression.

As CHST11 was the most upregulated GAG biosynthetic enzyme observed in COPD patients compared to controls, we performed gene set enrichment analysis on distal lung tissue that indicated that TGF-β targets were enriched in the dataset which indicated that TGF-β signaling could explain this increase. Moreover, we found CHST11 and other CS/DS biosynthetic enzymes to be positively correlated with the TGF-β expression profile in this patient cohort. The activity of TGF-β in COPD is not altogether clear as it may vary with disease severity and most studies have used tissues and plasma from patients during stable disease, while less is known during exacerbations. TGF-b is expressed in most compartments in of COPD patients and controls^18^.

By immunohistochemistry it was shown that CHST11 is expressed in several structural cells including fibroblasts and alveolar type II cells. Fibroblasts and also alvolar type II cells have an important role as producers of extracellular matrix (ECM) components in lung homeostasis^19,20^. Dysregulation of the ECM due to a derailed production and/or degradation is central in chronic lung diseases including pulmonary fibrosis and COPD. We have previously shown that distally derived lung fibroblasts from COPD patients have altered proteoglycan production compared to control subjects with increased levels of the CS/DS proteoglycan versican and decreased levels of the HS proteoglycan perlecan^21^. In addition, TGF-β stimulation increased the production of two of the most abundant CS/DS proteoglycans, versican and biglycan, 2- and 3-fold, respectively. The TGF-β induced increase in biglycan was confirmed in the current study by LC–MS. This suggests that in COPD proteoglycan production is altered in distal lung and that TGF-β stimulates the production of CS/DS bearing proteoglycans. COPD is characterized by increased turnover of the ECM and remodelling in distal airways that results in emphysema that has been suggested to be caused by an imbalance of protease and antiprotease activity^22^. Besides neutrofil elastase, matrix metalloproteinases have been suggested play a major role^23^. CS/DS has been demonstrated to be involved in the activation of MMP-2, MMP-13, ADAMTS4, ADAMTS7B and may thus contribute to the imbalance^23–25^.

Increased levels of HS and CS/DS have been observed in bronchoalveolar lavage fluid from COPD patients during acute exacerbations and this was correlated to airway obstruction^26^. There is also other evidence of the influence of sulfated CS/DS in COPD such as role of sulfatase modifying factor (SUMF1), which removes sulfate groups from CS/DS chains. Polymorphism in the SUMF1 gene has been reported in COPD patients and this was associated with lower mRNA levels in sputum and increased sulfation of CS/DS and worsening of the COPD conditions^27^. The observed structural changes of CS/DS and HS in COPD patients are crucial for many biological properties, such as cytokine storage of FGF, HGF control of coagulation, migration and activation of collagen fibril formation^8,28–33^. Quantitative changes in GAGs may either be due to changes in the polymer-chain lengths or in the amount of core proteins. In this study, a dataset that compared the matrisome proteome in distal lung samples from COPD Gold stage IV patients and controls from a previous study was re-analyzed^1^. As expected, the most abundant CS/DS proteoglycans were biglycan and decorin, Collagen XII, Prolargin and the plasma proteoglycan Bikunin but there were no significant differences between controls and COPD patients indicating that the increase in CS/DS may be either due to the number of GAG-chains or in increased chain lengths rather than in the amount of proteoglycans^34^. However, the cohorts were relatively small and the patients were all in GOLD stage IV, and it cannot be excluded that there may be a clearer difference in milder disease. It has been shown that severe-early onset COPD patients have a unique fingerprint of ECM proteins, including several proteoglycans, compared to older patients with moderate disease. This suggests that both age and severity may influence the ECM remodeling in COPD^35^.

This study was based on lung tissue from different cohorts, and the COPD stage division was based on spirometry. This may be a limitation as the type and extent of remodeling may be structurally different. Tissue from control subjects and GOLD stage IV patients were from lung explants, while tissues from control smokers and GOLD stage II-III patients were lung cancer patients undergoing resections (lobectomies). The matrisome-proteome were done on small cohorts, which may have influenced the ability to detect differences. The immunohistochemistry to identify CHST11-positive cells was performed on tissue from one COPD patient and one control subject which is a limitation and bigger cohorts may have revealed interindividual and more disease specific differences. HFL1 cells were used to validate the response to TGF-b in an in vitro setting. These are primary cells derived from fetal lung tissue that have many characteristics of primary cells isolated from patients, but it cannot be excluded that they may differ in this specific response. Future studies will include validation in primary fibroblasts from COPD and non-COPD donors to assess clinical relevance and inter-individual variability.

In conclusion, in this study we show that HS and CS/DS were increased in distal lung tissue from COPD patients compared to controls and control smokers. In addition, there was an increase in *4-O-*sulfation in CS/DS in COPD patients and this was accompanied by increased expression of CHST11, one of the enzymes that modulates this specific modification. Finally, gene set enrichment and cell experiments indicated that CHST11 is likely induced by TGF-β signaling. Our findings may open new avenues for targeted therapeutic interventions to modulate CS/DS sulfation with the aim to limit tissue damage and disease progression in COPD.

## Material and methods

### Patient cohorts

The patient cohorts and in which analysis they have been used is summarized in Table 1.

**Table 1:** Patient cohort size in the study and number of subjects in each analysis.

	Group size	PG MS analysis	GAG analysis	IHC	RNA seq distal lung
*Cohort 1*					
Controls	11	5	11	1	
Control smokers	11		11		
COPD (GOLD II–III)	11		11		
COPD (GOLD IV)	11	5	11	1	
*Cohort 2 (GSE57148)*					
Controls	91				91
COPD (GOLD I–II)	98				98

#### Cohort 1

Lung explants from healthy organ donors (n = 11), with no history of cigarette smoking or lung disease were included in the study (Table 2). These lungs were either planned for clinical transplantation but were evaluated to not meet clinical grade upon arrival or were consented for research when there was a lack of matched recipients. Written consent was obtained from their closest relatives. Tissues from healthy smokers (n = 11) and COPD GOLD stage II-III patients (n = 11) were collected from tumor resections performed at Lund University hospital. Radiotherapy and chemotherapy previous to the surgery was an exclusion criteria. COPD patients had a postbronchodilator FEV1/FVC ratio of less than 0.7 and the healthy smokers had normal spirometry with FEV1/FVC ratio > 0.7 (Table 2). Tissue that was used in the study was taken as far away from the tumor as possible and was evaluated to be macroscopically healthy by an experienced pathologist. In addition, tissue was histologically assessed by hematoxylin and eosin (H&E) staining to ensure the absence of infiltrating cancer cells, but no immunohistochemical staining for markers such as desmin or PDGF-R was performed. Tissue from GOLD stage IV patients (n = 11) were from lung explants from transplantations performed at Sahlgrenska University hospital in Gothenburg. All donors provided informed consent, and the study was performed in accordance with the Declaration of Helsinki. Ethical approval for the study was obtained from the Swedish Ethical Review Authority (ethical permit numbers: 91-2006, 675-12-2012, 1026-15 2008-413), and all experiments complied with the relevant guidelines.

**Table 2:** Clinical characterization of COPD patients and control subjects in cohort 1.

	Non-smokers	Smokers	COPD (GOLD II-III)	COPD (GOLD IV)
No	11	11	11	11
Age (range)	59 (39–86)	66 (36–81)	69 (51–77)	61 (52–69)
Gender (M/F)	5/6	2/9	5/6	2/9
Pack years	0	28	41	30
Smoking status (Current/ex)	-	1/10	1/10	0/11
FEV_1_ (L)	N.D	2.21	1.95	0.85
FEV_1_ (%predicted)	N.D	88.9	66.8	28.7
FEV_1_/FVC	N.D	0.73	0.57	0.39
DLCO (%)	N.D	75	69	30

N.D. Not determined.

#### Cohort 2

The previously published RNAseq dataset, GSE57148, was generated from patients who required resection for lung cancer^36^. The inclusion criteria for COPD patients (n = 98) were a postbronchodilator FEV1/FVC ratio of less than 0.7 while controls (n = 91) had normal spirometry with FEV1/FVC ratio > 0.7. The average FEV1/FVC ratio for the COPD group was 0.57 and FEV1 predicted was 72% indicating that the majority of patients were in early stage (GOLD I-II). All study subjects were male and the mean age was higher in the COPD group compared to the control group, 67.5 years and 60.9 years, respectively. The mean pack years was also higher in the COPD group compared to the control group, 48.0 and 35.2 respectively.

### Material

HFL1 cells were obtained from ATCC (American Type Culture Collection). TGF-β1 was obtained from R&D, Minneapolis, MS, US. Heavy peptides för Selected Reaction Monitoring (SRM) analysis were bought from Innovative Peptide Solution, Berlin, Germany (Supplemental Table 1).

### Tissue dissection

After crude dissection, tissue pieces from distal/alveolar part of lungs were kept in Dulbecco’s MEM supplemented with 10% FBS, Gentamycin and Penicillin–Streptomycin (Gibco BRL, Paisley, UK). The samples were taken from just below the pleura and visible airways and vessels and pleura were systematically avoided. All samples were then stored in −80 °C until further analysis.

### Preparation for mass spectrometry

Tissue preparation and liquid chromatography-mass spectrometry (LC–MS/MS) analysis was done as described by Åhrman et al.^1^. In short, the tissue specimens were first homogenized resulting in a soluble fraction. Detergent soluble proteins were next extracted by boiling the remaining pellet in sodium dodecyl sulfate (SDS) buffer. Proteins were separated and washed on SDS-PAGE gels to remove SDS. The detergent resistant tissue remains were finally re-suspended in 8 M urea, reduced, alkylated, digested and digested with Lys-C and trypsinated to generate an ECM-enriched fraction.

### LC MS/MS, data analysis and assay library generation

LC–MS/MS analyses were performed on a Q-Exactive Plus mass spectrometer (Thermo Fisher Scientific). Peptides were separated on an EASY-nLC 1000 HPLC system connected to an EASY-Spray column and separated with a gradient elution. For data-dependent acquisition (DDA) full MS survey scans were performed. Data acquired in DDA and analyzed as described earlier^1^. For data-independent acquisition (DIA) MS survey scans at mass range 400–1200 m/z were followed by 32 MS/MS full fragmentation scans^37,38^. Data acquired in DDA were converted to mzML using MSconvert. All data analyses were managed in openBIS^39^. MS searches were performed using X! Tandem and OMSSA towards a human protein reference database (PTHR100) with reversed decoys. Resulting files were further analyzed in the Trans-proteomic pipeline (TPP v4.7POLAR VORTEX rev 0, Build 201405161127) using peptide Prophet, iProphet and MAYU^40,41^. The assay library used for DIA quantification was created according to the workflows included in openBIS^42^. Briefly, target assays were generated using spectraST, FDR calculations of 1% for peptide and protein were calculated with CLI and feature alignment with TRIC^43^. DIA files were analyzed using openSWATH^44^.

### Proteoglycan analysis with single-reaction monitoring (SRM) using mass spectrometry

Proteoglycans (PGs) were purified from cell medium using DE-52 columns. equilibrated with 6 M urea and 50 mM sodium acetate, pH 5.8. After application of sample, columns were washed with 6 M Urea, 50 mM acetate, 0.2 M NaCl buffer, pH 5.8. PGs were eluted with 6 M urea, 50 mM acetate, 0.8 M NaCl buffer with pH 5.8. Samples were desalted by PD-10 columns and freeze-dried. Freeze-dried proteins were re-suspended in 8 M urea, 100 mM ammonium bicarbonate pH 7.8, reduced with 5 mM Tris (2-carboxyethyl) phosphine (TCEP, Sigma-Aldrich) for 30 min at 37 °C at 800 rpm and alkylated with 10 mM iodoacetamide (Sigma-Aldrich) for 45 min at room temperature. Samples were diluted with ammonium bicarbonate to 1.6 M urea and digested with trypsin 1/100 w/w (Promega) overnight at 37 °C, 800 rpm. Samples from the soluble and ECM-enriched fractions were purified on C18 reversed phase spin columns (Harvard apparatus) prior MS analysis as described in^1^. Samples were re-suspended in buffer A (2% acetonitrile, 0.2% formic acid) with SRM peptides. The peptides together with 31.25 fmoles of heavy peptides (Supplemental material Table 1) were applied onto a pre-column (Zorbax 300SB- C18 5 × 0.3 mm, 5 µm, Agilent Technologies, Wilmington, DE). The peptides were then separated on a RPLC column (Zorbax 300SB-C18 150 mm × 75 µm, 3.5 µm, Agilent Technologies) and finally eluted with 95% solvent A in 0.1% FA in water and 40% solvent B in 0.1% FA in ACN. The hybrid Orbitrap – LTQ (Linear Ion Trap) XL mass spectrometer (Thermo Electron, Bremen, Germany) was operated in data dependent mode. Data was analyzed using Skyline v1.117 using the heavy peptides^45^.

### RNA sequencing (RNAseq) data analysis

A previously published RNAseq dataset were obtained from the Gene Expression Omnibus (GEO). The dataset was obtained from lung homogenates of 98 COPD (GOLD stage I-II) patients and 91 subjects with normal spirometry (GSE57148). Differential expression analysis was done using the package DESeq2 in the statistical software R when count data was available, otherwise, differential expression of transformed FPKM data was done using limma. Data visualization was done using the package ggplot2 and data expression boxplots are shown in Fragments per Kilobase of transcript per Million mapped reads (FPKM). Gene set enrichment analysis (GSEA) was done using the package clusterProfiler using ranked lists from the differential expression of each dataset, gene sets used for enrichment are referenced in each plot. To evaluate correlations between GAG enzymes and TGF-β signaling, a gene set variation analysis (GSVA)–based scoring and subsequent correlation framework were implemented in R. TGF score was computed per sample in the RNAseq dataset (GSE57148) by evaluating pathway enrichment scores of the normalized gene expression using the R package GSVA. Subsequently, Pearson and Spearman correlation analyses of log₂ transformed expression values of each gene of the GAG enzymes against the TGF-β scores. Correlation coefficients and corresponding p-values are computed and displayed on scatter plots generated with *ggplot2*. Code used to generate plots is deposited in GitHub Repository (https://github.com/Alsafadi/COPD_GAGs).

### Cell culture

Human Fetal Lung fibroblast 1 (HFL1) cells were cultured in Minimum essential Medium (MEM) supplemented with 10% FBS, 1% Penicillin Streptomycin (PEST) and 1% Glutamine at 37° in 5% CO_2_. Cells were treated with 5 ng of TGF-β /ml in MEM supplemented with 0.4% FBS for 48 h. We selected a well-established primary cell line from human fetal lung instead of using primary cultures from COPD and non-COPD patients that is highly heterogenous due to disease stage, patient age, and the location within the lung from where they were isolated^46^.

### Glycosaminoglycan disaccharide analysis

A minimum of two separate tissue pieces was analyzed from each individual. The method to isolate, digest and analyze GAG disaccharide has been developed in our laboratory which previously has been described in detail^1,47,48^. All tissue samples were first lyophilized and weighed. Approximately 2 mg tissue (dry weight) was used from each tissue sample. Proteins were degraded by an overnight incubation with pronase and benzonase. GAGs were purified on an anion spin column (Vivapure Q mini H, Sigma-Aldrich, St. Louis, MO, USA). Samples were desalted and then subjected to chondroitinase ABC or chondroitinase B digestion which generated disaccharides from CS/DS and DS, respectively. Heparan sulfate was degraded with a mixture of recombinant heparinase I, II and III (a kind gift from Jian Liu, University of North Carolina at Chapel Hill). Fluorophore-labelling of the resulting disaccharides was performed by incubation with 2-aminoacridone (AMAC) (Sigma-Aldrich, St Louis, MS, USA) followed by addition and incubation with NaBH3CN. The labelled disaccharides were separated on HPLC-fluorescence on a X-Bridge BEH Shield RP18 (2.1 × 100 mm, 2.5 m) column connected to a Thermo Scientific UltiMate 3000 Quaternary Analytical system with an FLD-3400RS fluorescence detector (excitation = 428 and emission = 525) using a gradient NH4OAc, 60 mM, pH 5.6, and 2% B: MeCN. Quantification was done by comparison to known weight of standard disaccharides (Iduron, UK) mock-treated in the same buffers and enzymes as the samples in each series of runs.

### Immunohistochemistry

4 μm cryosections of formalin-fixed, paraffin-embedded lung tissue sections were rehydrated with xylene and ethanol according to standard procedures. Heat activated antibody retrieval was performed at pH 6.1 using DAKO PT link (Dako, Agilent Technologies, Santa Clara, CA, USA) followed by endogenous peroxidase blocking and blocking with normal goat serum. Sections were incubated with primary antibody (1:200, Anti-CHST11, #HPA052828, Atlas antibodies, Stockholm, Sweden) for 1 h followed by incubation with labelled polymer-HRP for 30 min. Sections were developed with DAB solution included in the kit Dako (EnVision Dual Link System-HRP (DAB +) K4065) and counterstained with Mayer’s hematoxylin (Sigma-Aldrich, St. Louis, MO, USA). Sections were photographed using an Olympus BX50F microscope. Tissue staining was performed on tissue from one control subject and one COPD GOLD stage IV patient. Staining specificity was controlled by omitting the primary antibody as shown in supplement Fig. S4. The identification of different cell types was based on location, size and morphology and no cell-specific co-staining was performed.

### RNA isolation, cDNA preparation and real-time quantitative PCR analysis

Cell pellets were harvested in RNAlater (Qiagen, Hilden, Germany) and stored in − 70 °C. Total RNA was isolated using the mini RNeasy Mini Kit from (Qiagen). For preparation of cDNA, the Quantitect Reverse Transcription Kit (Qiagen) was used. For the qPCR, cDNA preparations were mixed with Quantitect SYBR Green (Qiagen) along with specific pre-designed and validated human primers from Qiagen: Chondroitin sulfate synthase 1, *CHSY1*,

Chondroitin sulfate N-acetylgalactosaminyltransferase 2, *CSGALNACT2*, Uronyl 2-sulfotransferase, *UST*, Carbohydrate sulfotransferase 3, *CHST3*, Carbohydrate sulfotransferase 7, *CHST7*, Carbohydrate sulfotransferase 11, *CHST11*, Carbohydrate sulfotransferase 12, *CHST12,* Carbohydrate sulfotransferase 13, *CHST13,* Carbohydrate sulfotransferase 14, *CHST14,* Carbohydrate sulfotransferase 15, *CHST15,* Dermatan-sulfate epimerase, *DSE*, and analyzed on a Mx3005P qPCR system (Stratagene, La Jolla, CA, USA) with standard cycling parameters to perform thermo-cycling and real-time detection of PCR products^48^.

### Statistical analysis

Data are expressed as mean ± SD. For the GAG analysis, non-parametric Kruskal–Wallis tests combined with Dunn’s multiple comparison test that corrects for multiple testing. For the cell experiments student t-tests were used. Differences were considered significant at *p* < 0.05. All analyses were performed using GraphPad Prism software. Statistical analysis of RNAseq data was done using the packages DESeq2 and limma and cut-offs for differential gene expression were set based on adjusted *p*-values.

## Supplementary Information

Supplementary Information 1

Supplementary Information 2

## Data Availability

The RNAseq dataset that was used were obtained from the Gene Expression Omnibus (GEO): (GSE57148). The used code to generate the data and the plots is deposited in GitHub Repository: (https://github.com/Alsafadi/COPD_GAGs).
