# Supplementary material for: Change in the chondroitin/dermatan structure in distal lung tissue from COPD patients
Source: Sci Rep. 2026 Mar 23;16:9721. doi: 10.1038/s41598-026-44120-4 (PMC13013573; doi:10.1038/s41598-026-44120-4)

**Supplemental material**

Supplemental table 1. Intensity of heavy peptides used for SRM quantitation

| **Peptide** | charge ++ | charge +++ |
| --- | --- | --- |
| Versican |  |  |
| LLASDAGLYR | 23466924 |  |
| Decorin |  |  |
| VPGGLAEHK | 1352573 | 1667573 |
| Syndecan 2 |  |  |
| VETTTLNIQNK | 6998996 |  |
| Glypican 1 |  |  |
| VNPQGPGPEEK | 2428670 |  |
| SHAELETALR | 2531400 | 1478018 |
| Biglycan |  |  |
| VPSGLPDLK | 6412563 |  |
| Aggrecan |  |  |
| YPIVSPR | 31933118 |  |
| Agrin |  |  |
| SELFGETAR | 25987664 |  |
| CD44 |  |  |
| FAGVFHVEK | 3258855 | 1490365 |
| Glypican 4 |  |  |
| NDAPLHEINGDHLK | 10591 | 177166 |
| CSPG 4 |  |  |
| ASEAVEDTFR | 13079808 |  |
| Collagen XII |  |  |
| TEFNLNQYYQR | 1369296 |  |
| Collagen XV |  |  |
| TADTAVTGLASPLSTGK | 6193447 | 52429 |
| Perlecan |  |  |
| FDAGSGMATIR | 15089664 |  |
| Glypican 6 |  |  |
| TFVQGLTVGR | 26297962 |  |
| Syndecan 1 |  |  |
| EGEAVVLPEVEPGLTAR | 2598369 | 53267 |

The intensity of 200 femtomole peptides injected for quantification of proteoglycans in the cell culture preparations.

**Supplemental Figure S1**. HPLC chromatogram of derivatized heparan sulfate disaccharides from representative control and COPD Gold stage IV patient.


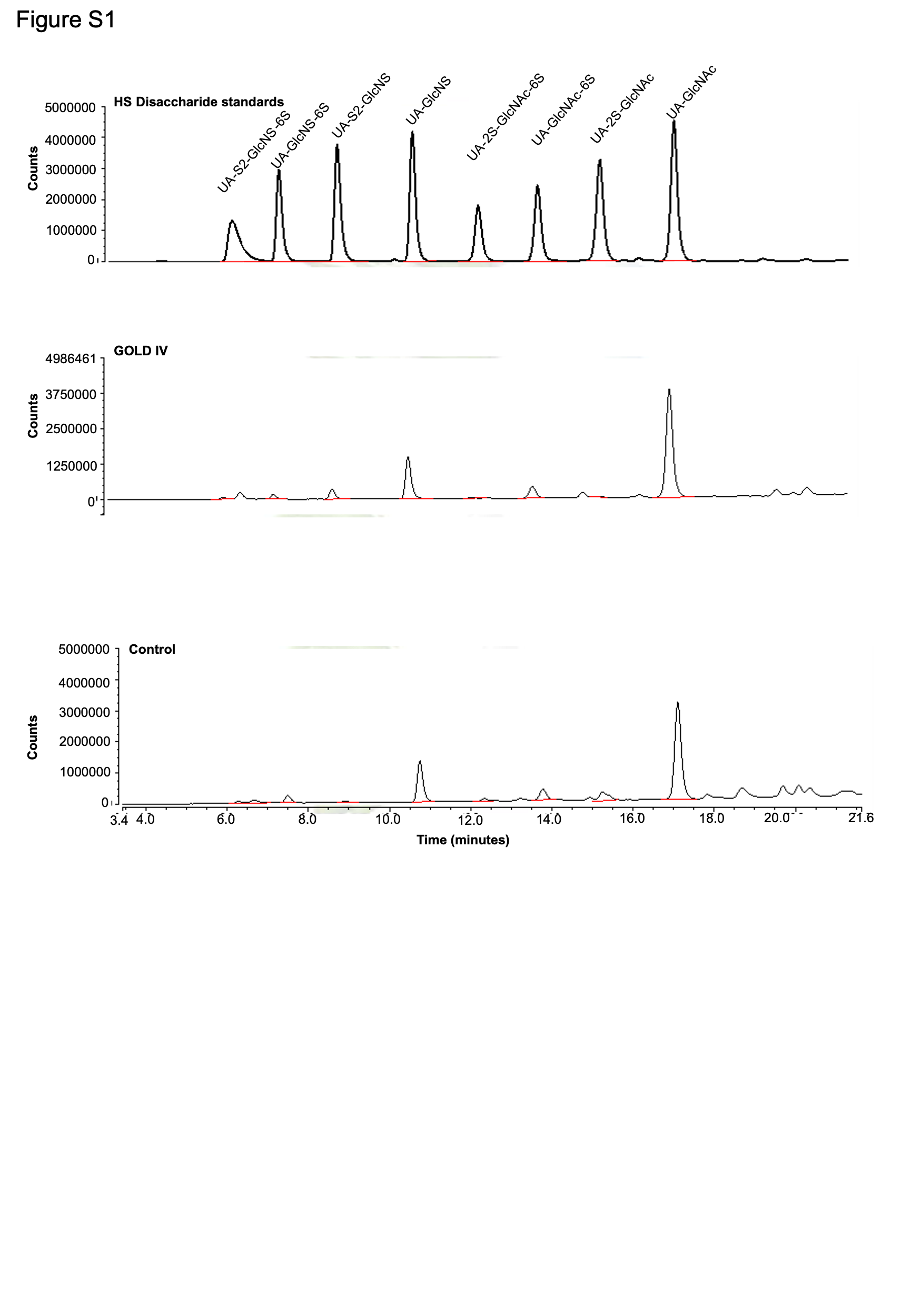


**Supplementary Figure S1 – Separation and quantification of HS disaccharides**

**Top panel** - elution of HS disaccharide standards. Heparinase I, II and III-generated disaccharides from distal lung tissue from representative COPD patient (**middle panel**) and control (**bottom panel**) were AMAC labelled and separated by RP-HPLC.

**Supplemental Figure S2**. HPLC chromatogram of derivatized chondroitin sulfate/dermatan sulfate/hyaluronan disaccharides from representative control and COPD Gold stage IV patient.


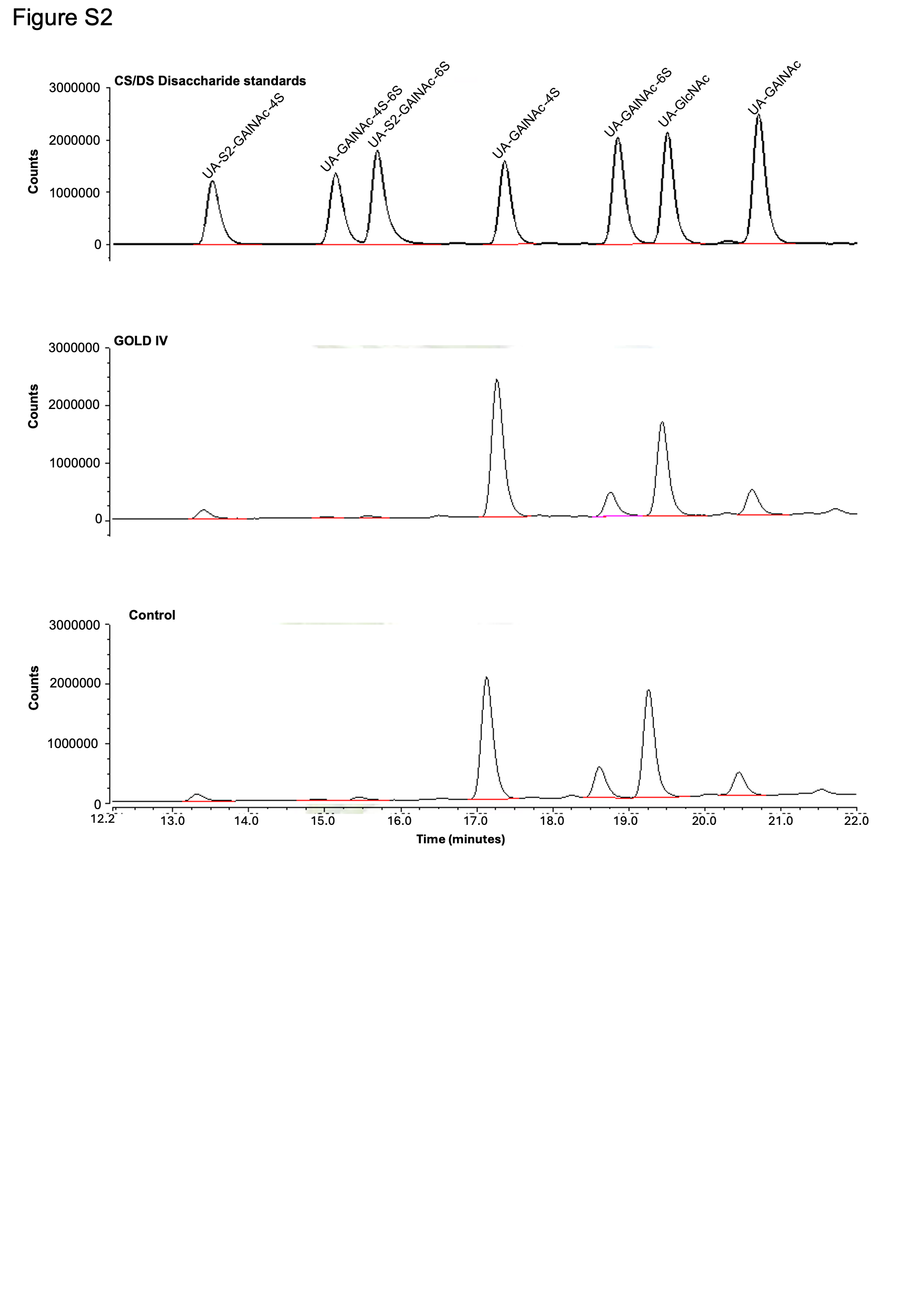


**Supplementary Figure S2 – Separation and quantification of CS/DS/HA disaccharides**

**Top panel** - elution of CS/DS/HA disaccharide standards. Chondroitinase ABC-generated disaccharides from distal lung tissue from representative COPD patient (**middle panel**) and control (**bottom panel**) were AMAC labelled and separated by RP-HPLC.

**Supplemental Figure S3** - Transcriptomics analysis of HS and CS/DS biosynthetic enzymes. P-values shown in plot titles are the adjusted p-value.

A. CS/DS related genes


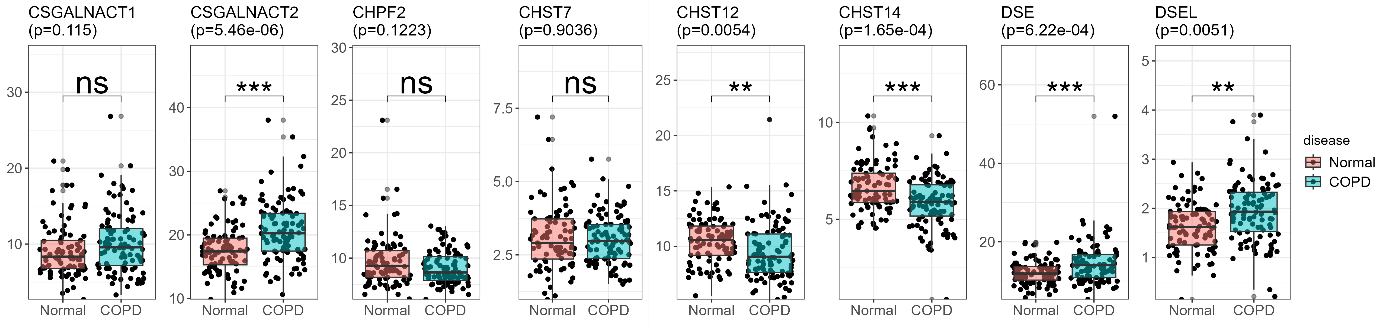


B. HS related genes


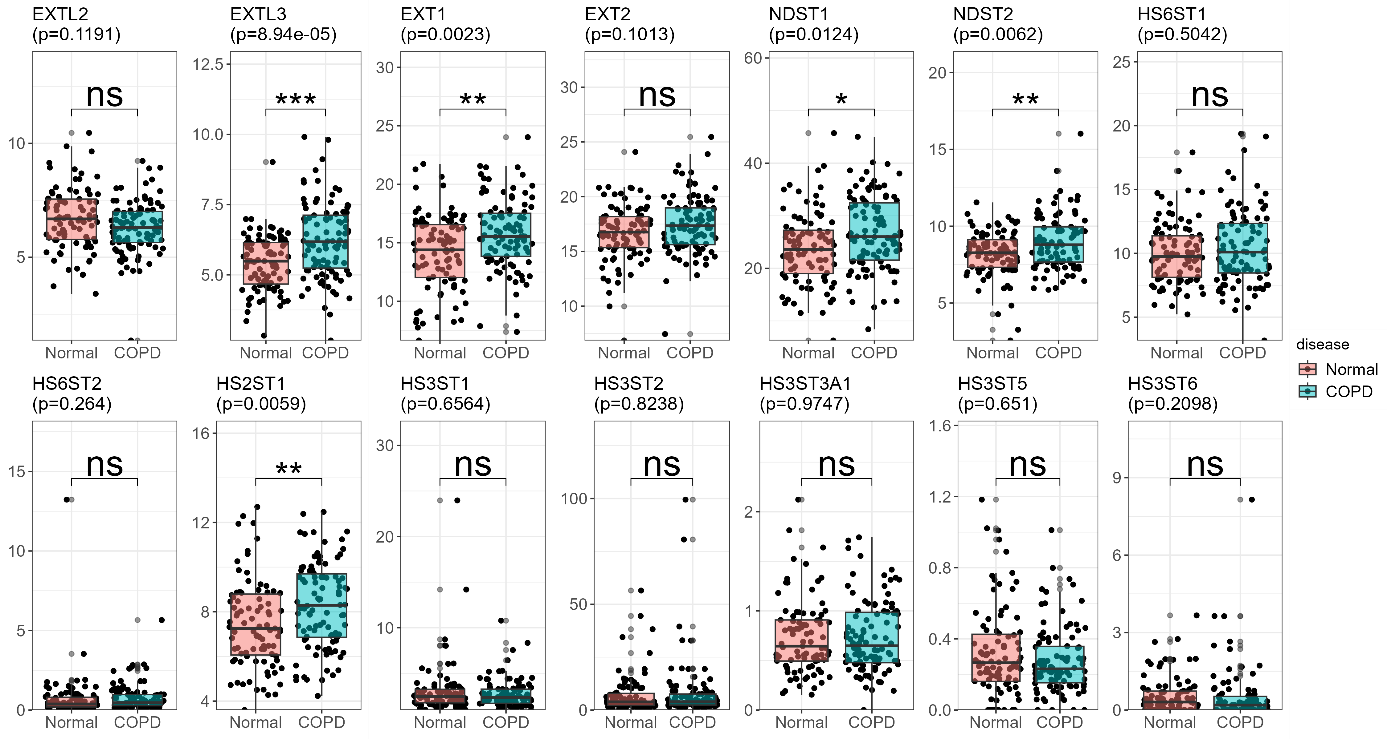


C. Core related genes


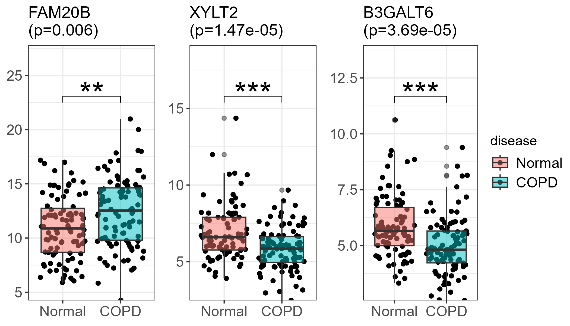


**Supplemental Figure S4** – Immunohistochemistry staining controls

A. Immunohistostaining of CHST11 (HPA052828), control subject


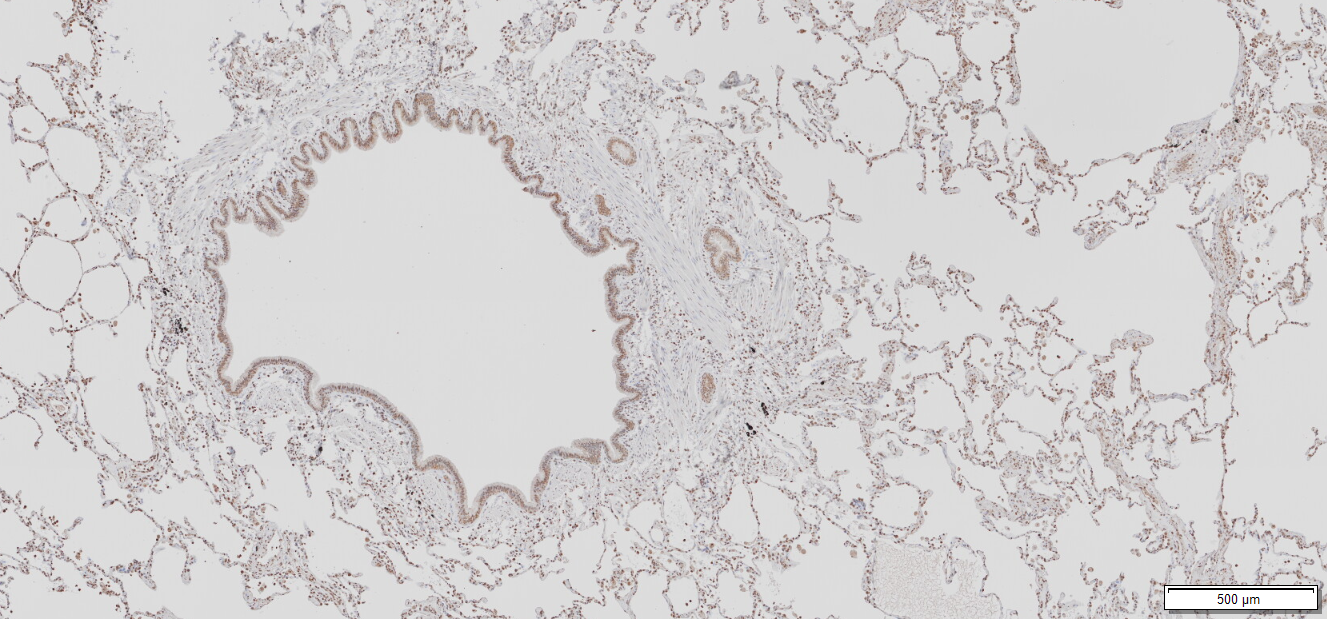


B. Immunohistostaining of CHST11 (HPA052828), control subject, primary antibody omitted


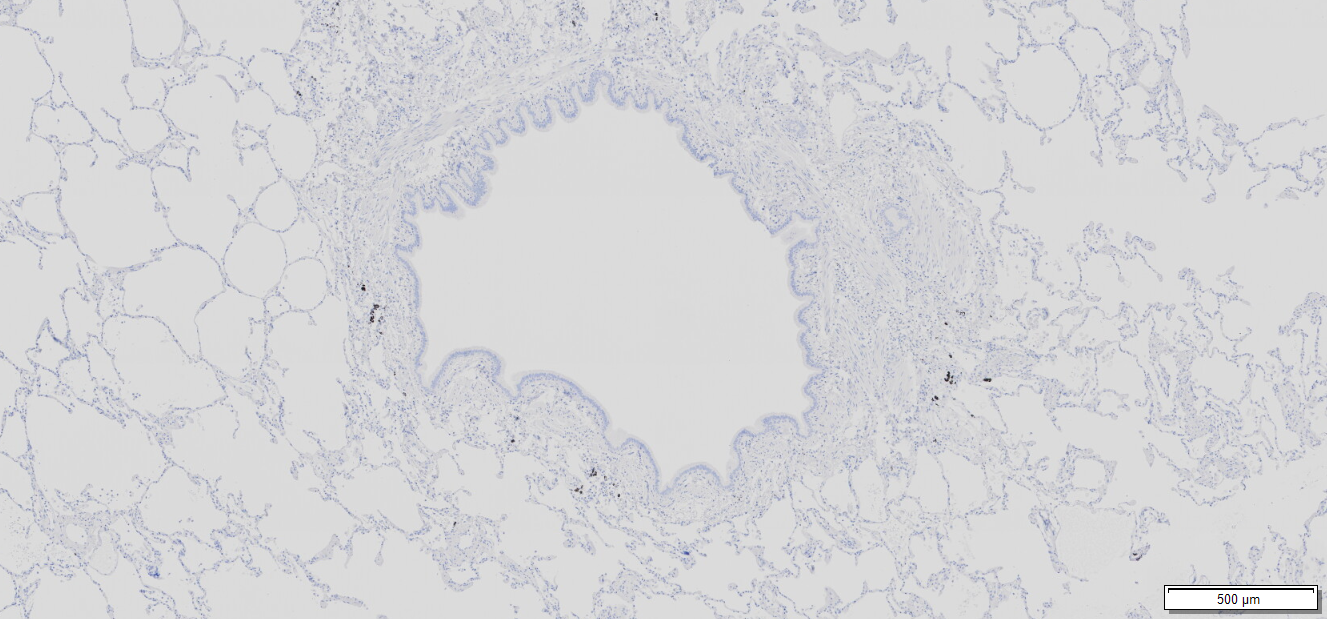


C. Immunohistostaining of CHST11 (HPA052828), COPD patient


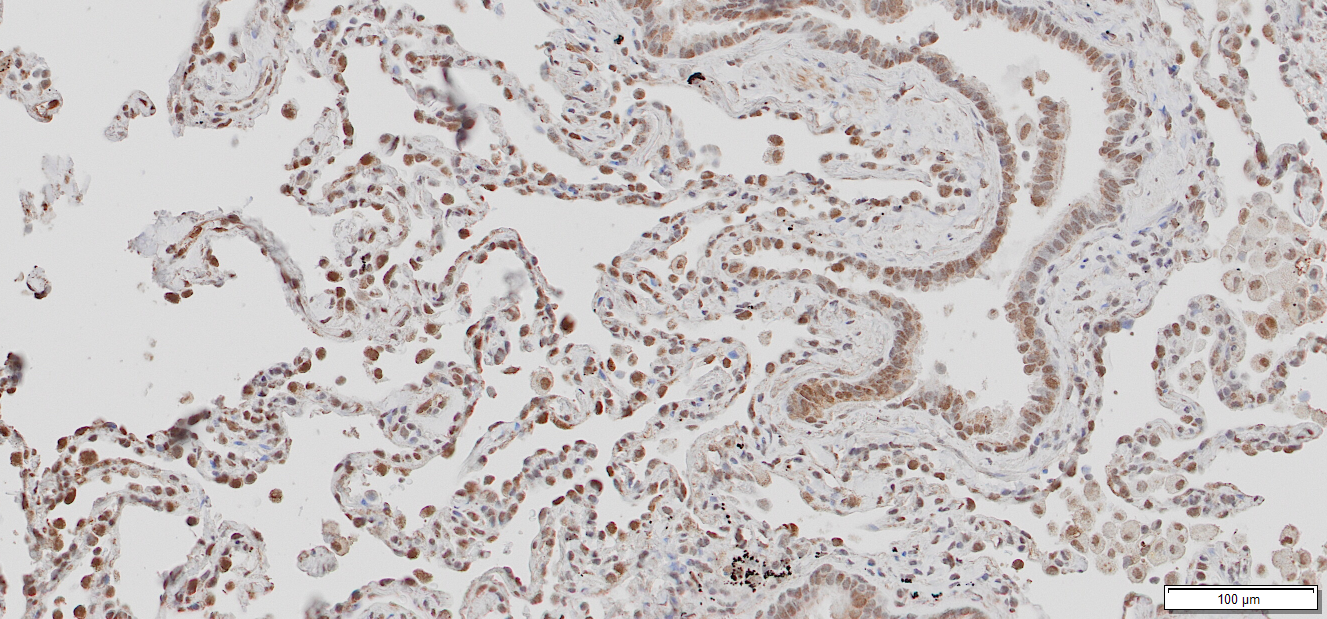


C. Immunohistostaining of CHST11 (HPA052828), COPD patient, primary antibody omitted


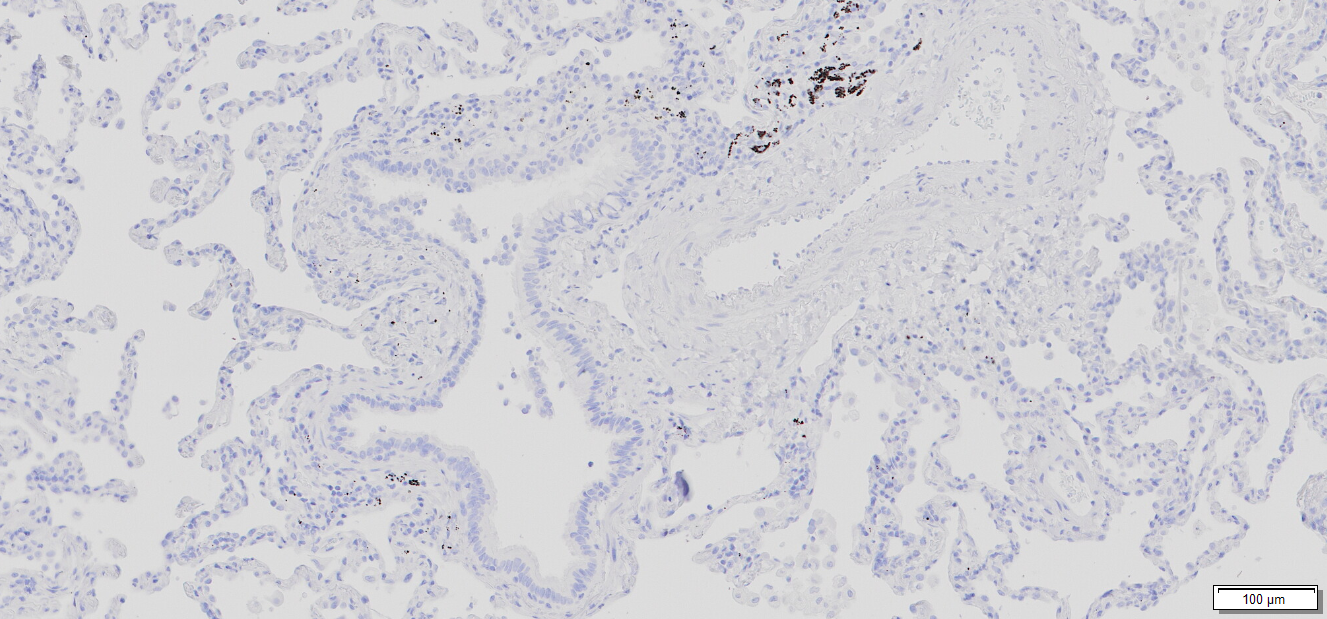


**Supplemental Figure S5** – Correlation Analysis of TGF-β signaling score with GAG enzymes.

1. CS/DS Genes


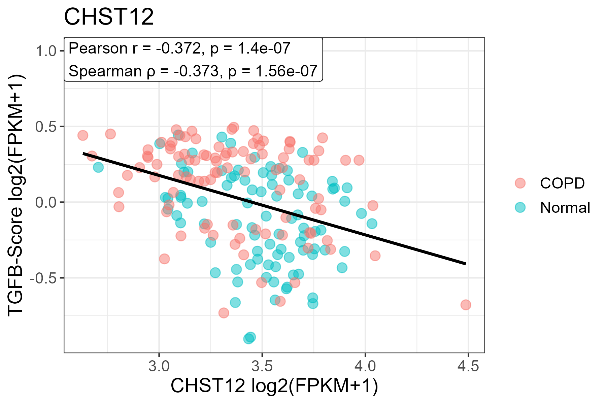

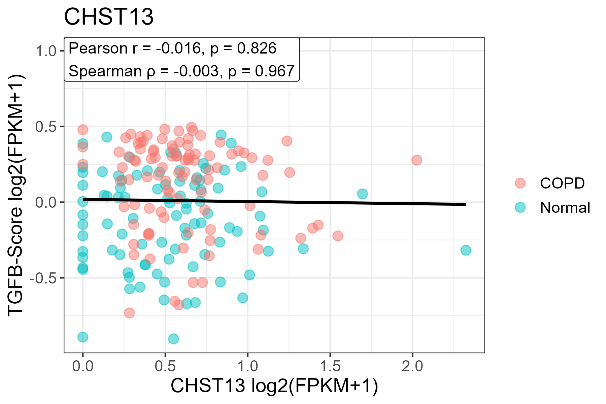

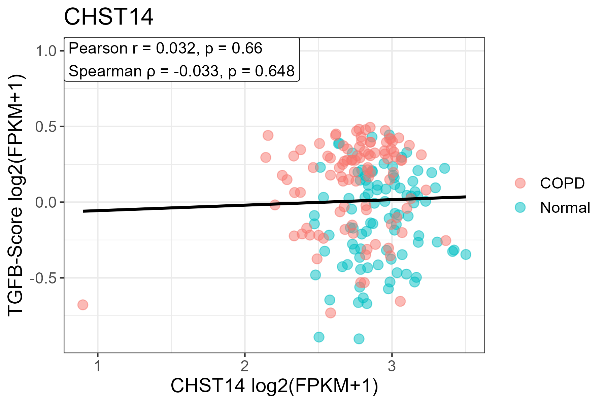

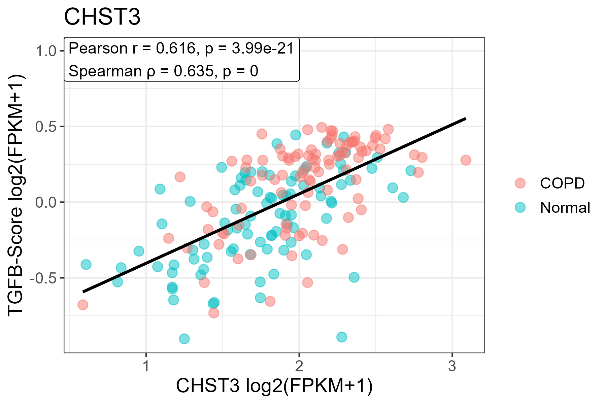

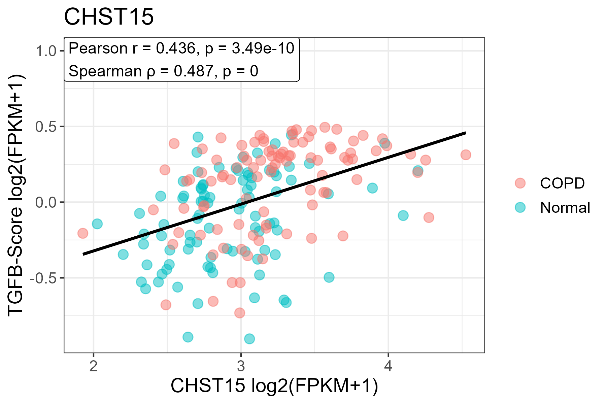

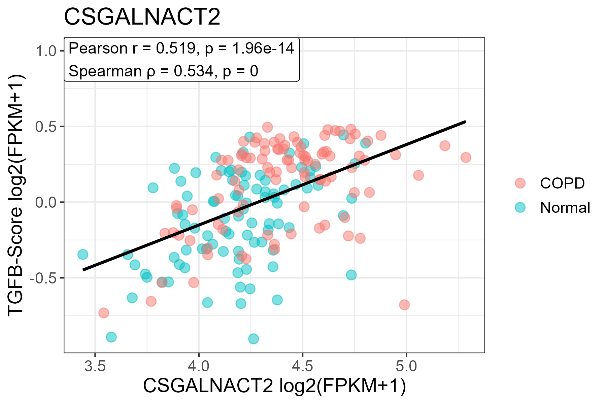

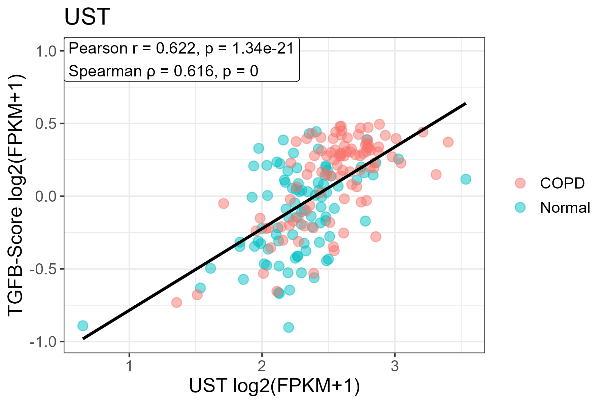

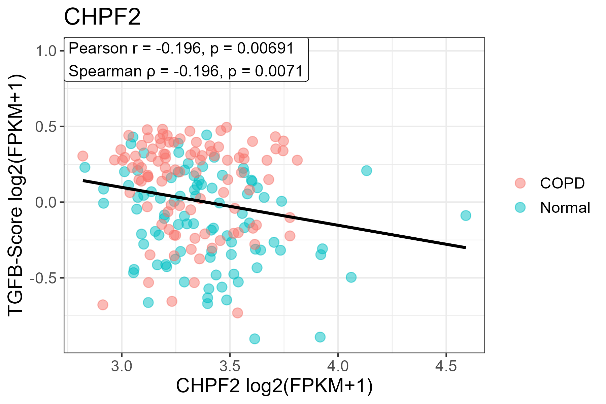

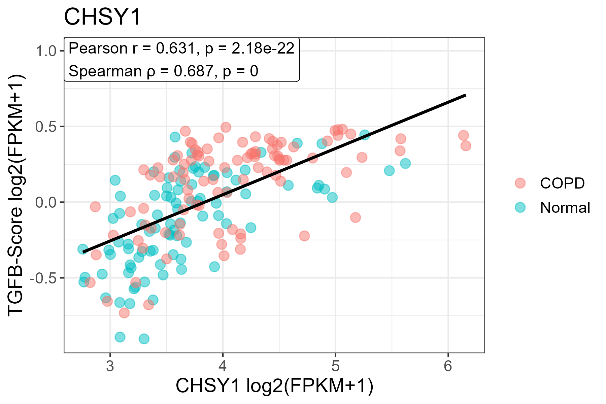

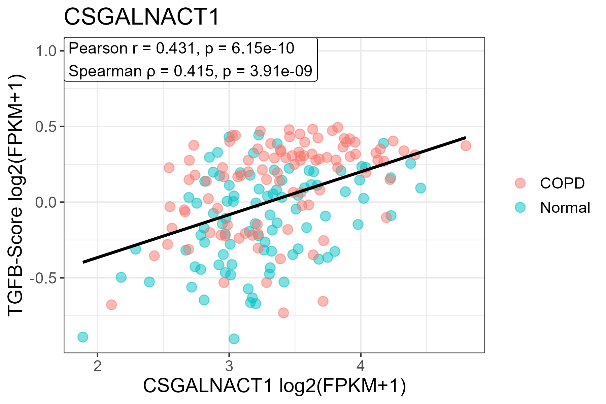

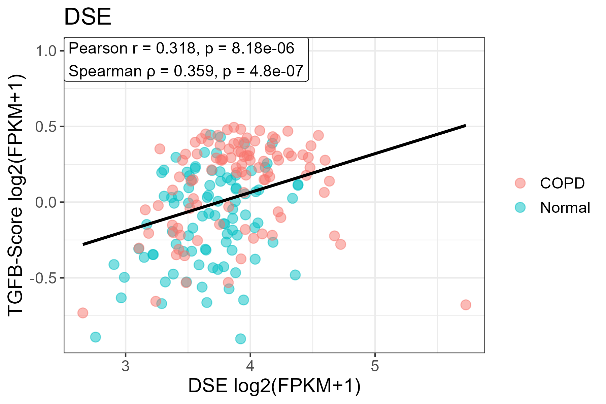

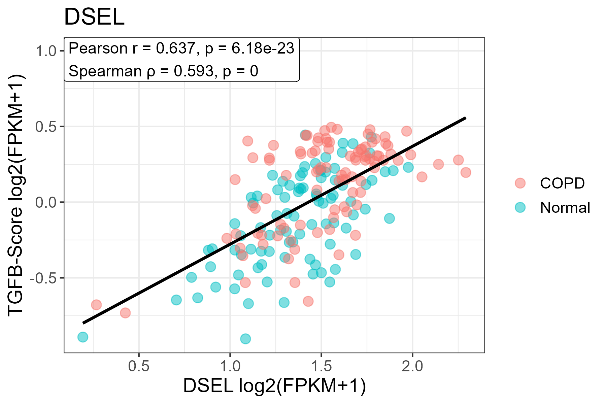

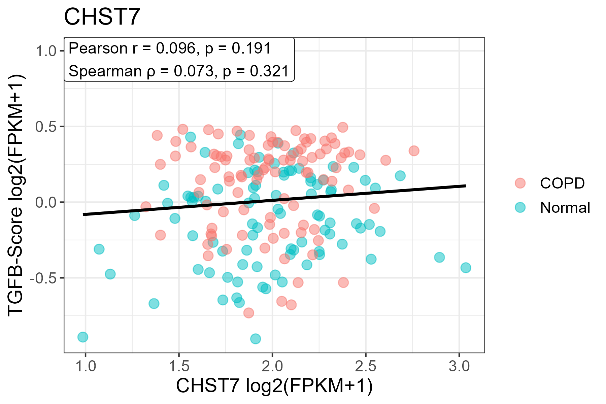


1. HS Genes


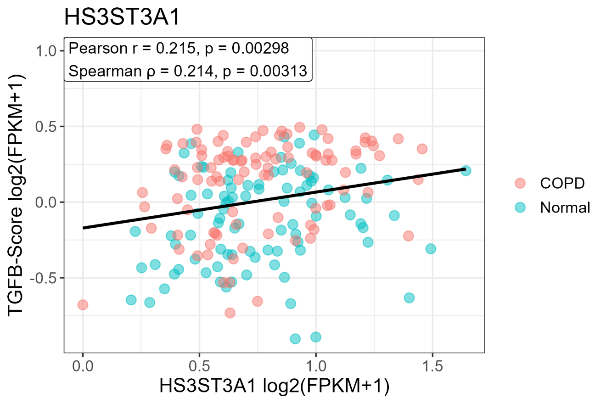

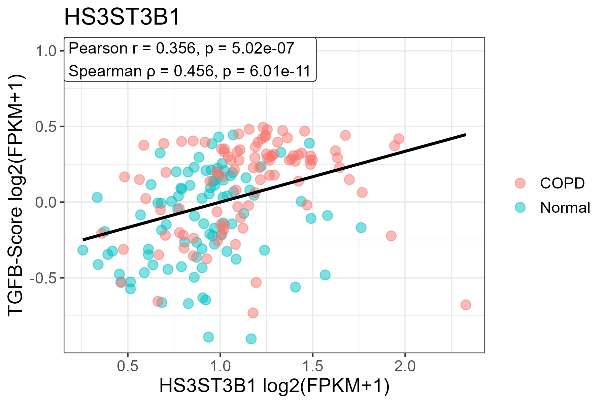

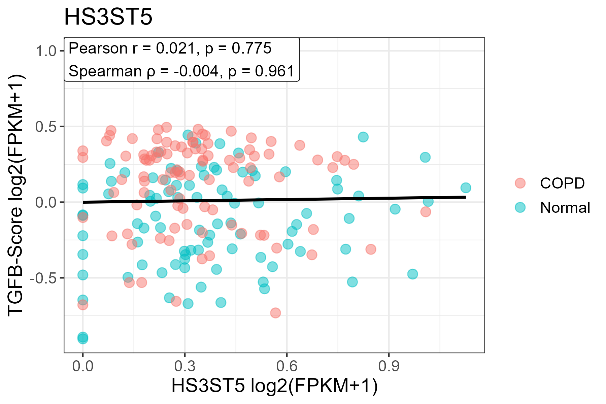

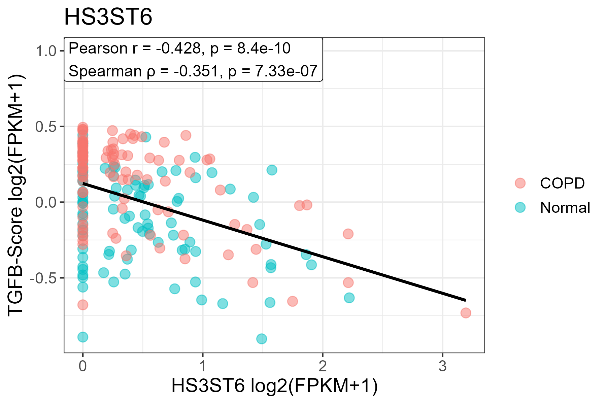

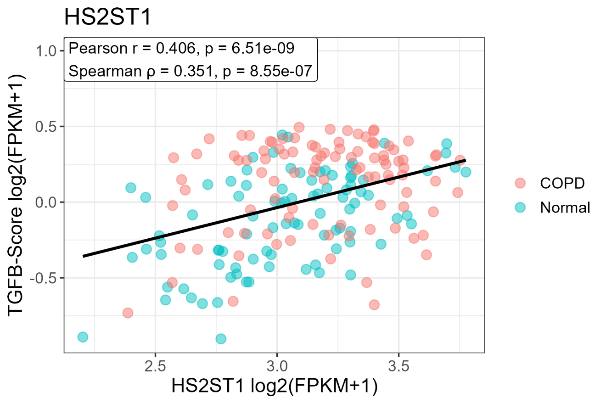

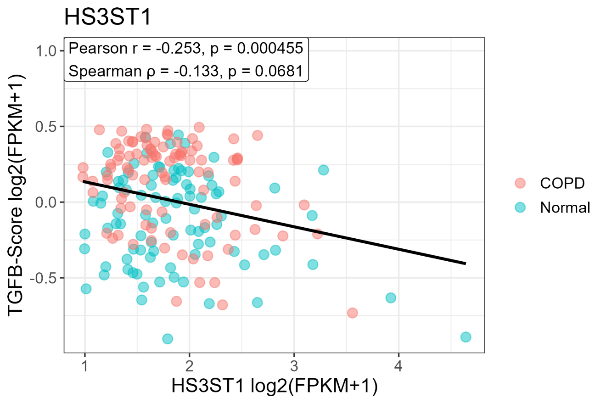

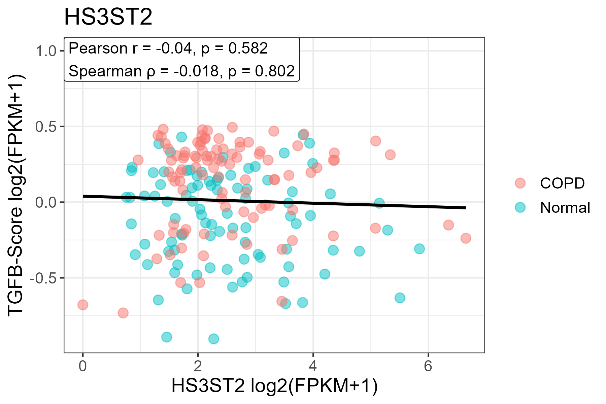

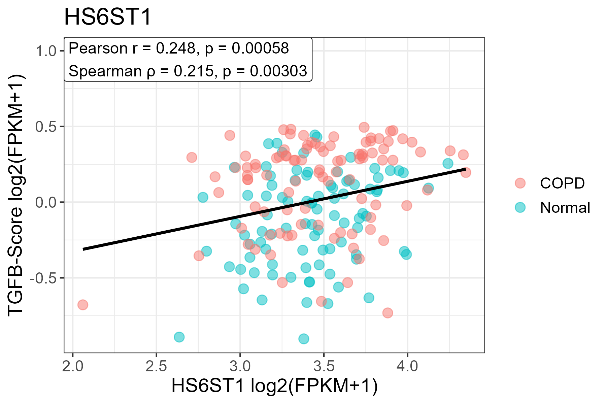

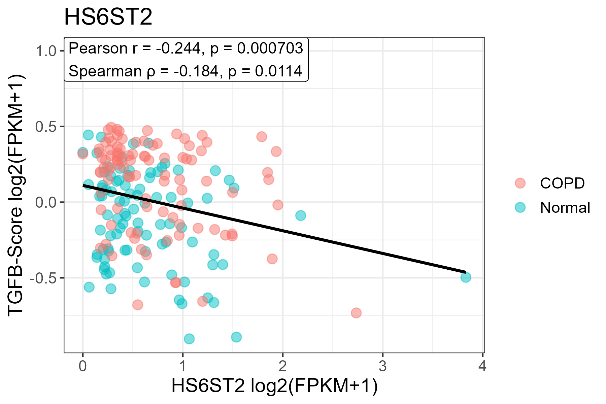

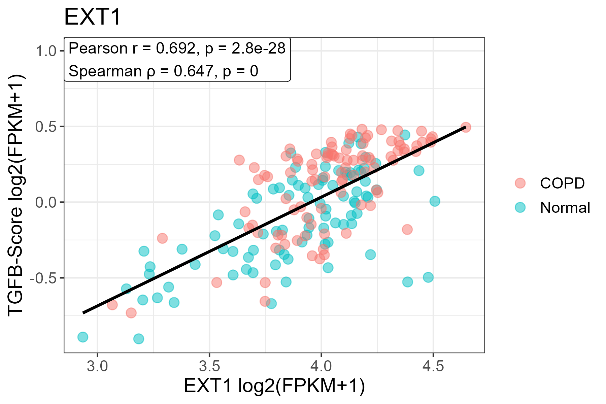

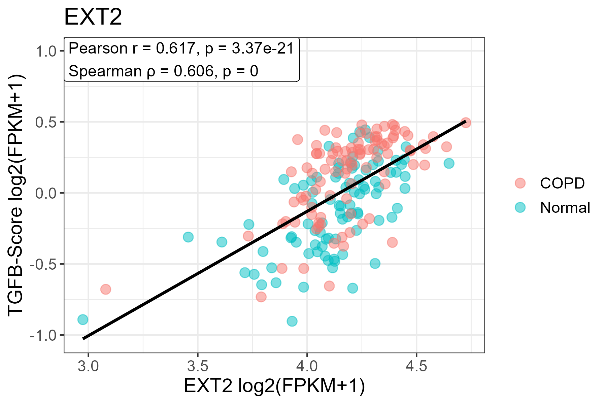

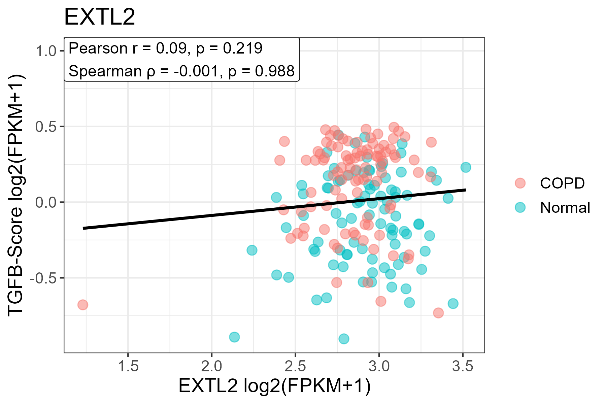

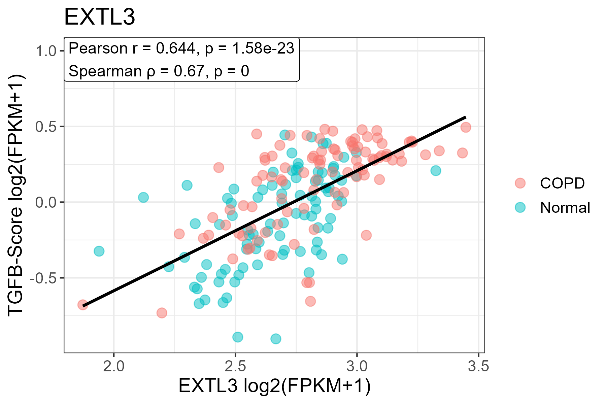

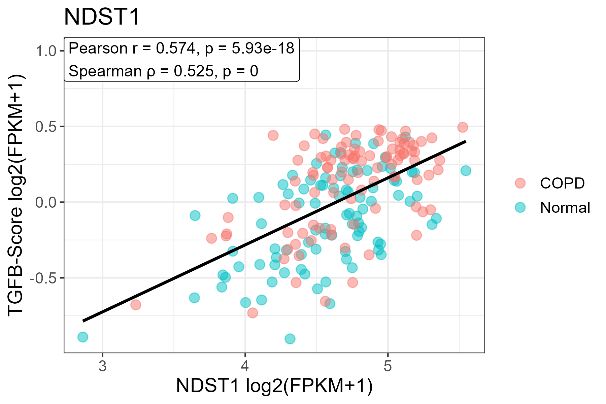

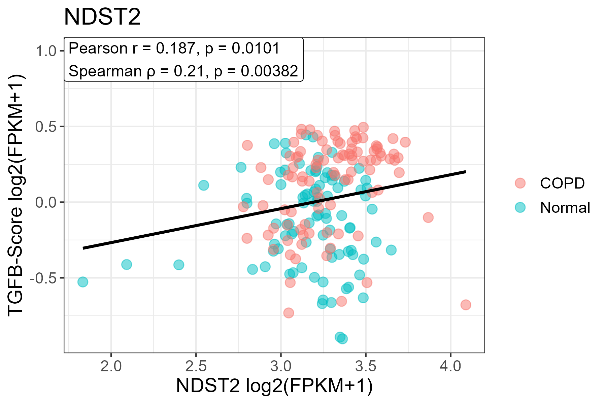


1. Core Genes


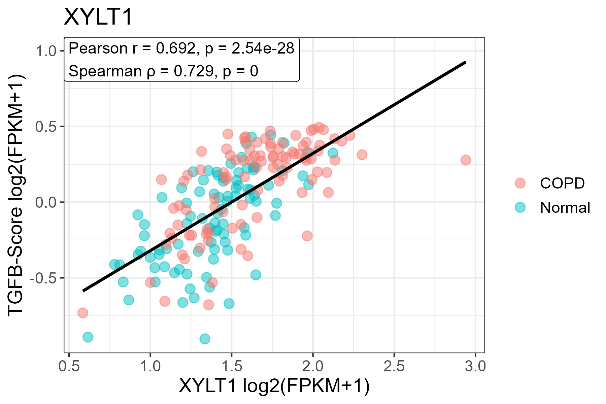

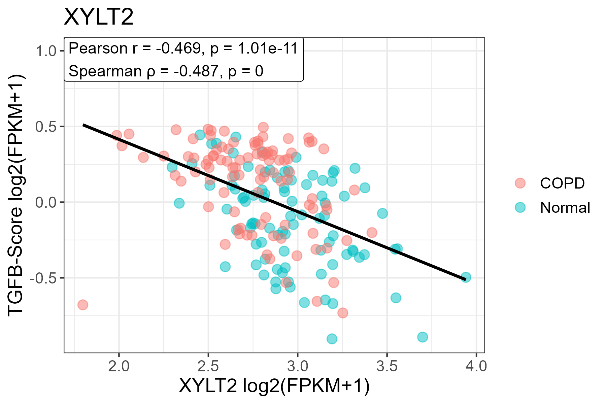

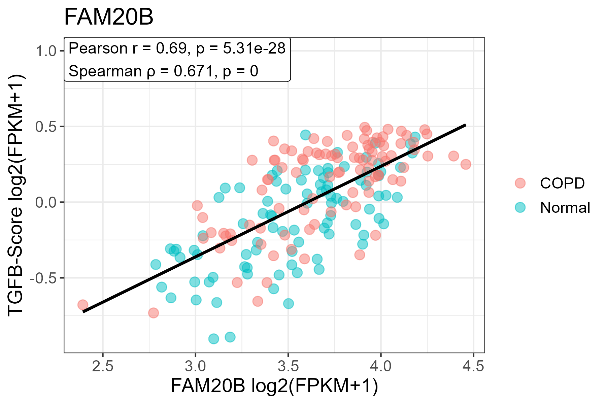

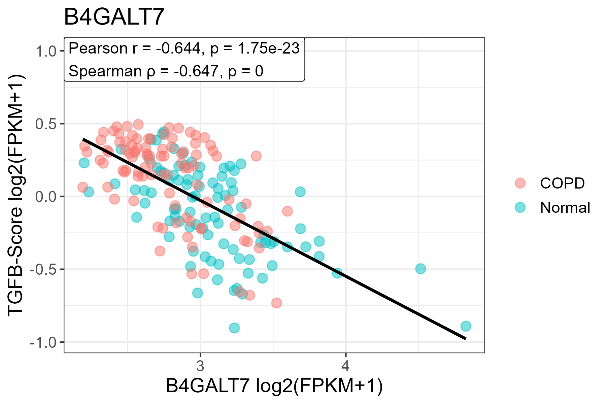

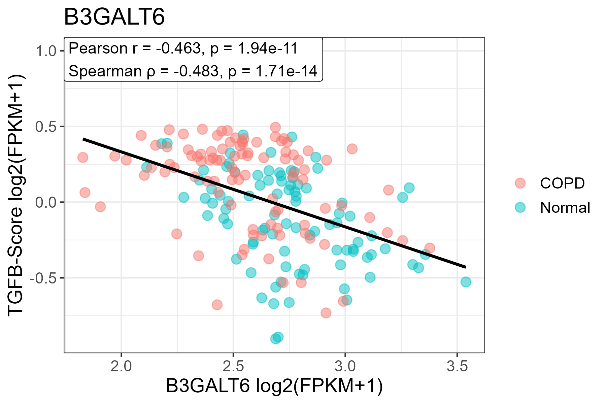

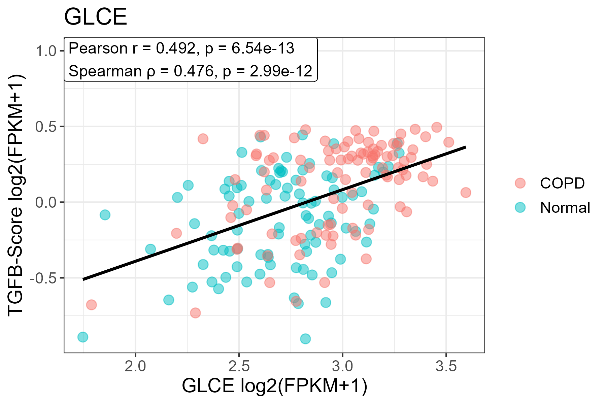

Supplement: Supplementary file 1 — Supplementary Information 1 [file 41598_2026_44120_MOESM1_ESM.docx]
